# Genomic structure and expression of *Jmjd6 *and evolutionary analysis in the context of related JmjC domain containing proteins

**DOI:** 10.1186/1471-2164-9-293

**Published:** 2008-06-18

**Authors:** Phillip Hahn, Jens Böse, Stefanie Edler, Andreas Lengeling

**Affiliations:** 1 Research Group Infection Genetics, Department of Experimental Mouse Genetics, Helmholtz Centre for Infection Research, D-31824 Braunschweig, Germany; 2 The Roslin Institute and Royal (Dick) School of Veterinary Studies, Easter Bush Veterinary Research Centre, The University of Edinburgh, Roslin, EH25 9RG, UK

## Abstract

**Background:**

The *jumonji C (JmjC) domain containing gene 6 *(*Jmjd6*, previously known as phosphatidylserine receptor) has misleadingly been annotated to encode a transmembrane receptor for the engulfment of apoptotic cells. Given the importance of JmjC domain containing proteins in controlling a wide range of diverse biological functions, we undertook a comparative genomic analysis to gain further insights in *Jmjd6 *gene organisation, evolution, and protein function.

**Results:**

We describe here a semiautomated computational pipeline to identify and annotate JmjC domain containing proteins. Using a sequence segment N-terminal of the Jmjd6 JmjC domain as query for a reciprocal BLAST search, we identified homologous sequences in 62 species across all major phyla. Retrieved *Jmjd6 *sequences were used to phylogenetically analyse corresponding loci and their genomic neighbourhood. This analysis let to the identification and characterisation of a bi-directional transcriptional unit compromising the *Jmjd6 *and *1110005A03Rik *genes and to the recognition of a new, before overseen *Jmjd6 *exon in mammals. Using expression studies, two novel *Jmjd6 *splice variants were identified and validated *in vivo*. Analysis of the *Jmjd6 *neighbouring gene *1110005A03Rik *revealed an incident deletion of this gene in two out of three earlier reported *Jmjd6 *knockout mice, which might affect previously described conflicting phenotypes. To determine potentially important residues for *Jmjd6 *function a structural model of the Jmjd6 protein was calculated based on sequence conservation. This approach identified a conserved double-stranded β^-^helix (DSBH) fold and a HxDx_n_H facial triad as structural motifs. Moreover, our systematic annotation in nine species identified 313 DSBH fold-containing proteins that split into 25 highly conserved subgroups.

**Conclusion:**

We give further evidence that *Jmjd6 *most likely has a function as a nonheme-Fe(II)-2-oxoglutarate-dependent dioxygenase as previously suggested. Further, we provide novel insights into the evolution of Jmjd6 and other related members of the superfamily of JmjC domain containing proteins. Finally, we discuss possibilities of the involvement of *Jmjd6 *and *1110005A03Rik *in an antagonistic biochemical pathway.

## Background

The jumonji C (JmjC)-domain containing proteins are an extending family of redox enzymes that catalyse a wide range of oxidation reactions. These proteins are found in all living organisms and are characterised by sharing the highly conserved JmjC domain. This domain was first described by Takeuchi and colleagues, who isolated a gene in the mouse which they called *jumonji *(meaning cruciform in Japanese), in regard to an abnormal phenotype visible in mutant embryos during neural plate development [1]. Since then, the JmjC domain has found its entry into public domain databases and many other proteins containing this domain have been annotated or identified subsequently [2-5]. Currently, more then 11,000 sequence entries of JmjC domain containing proteins are present in Uniprot, PFAM, Interpro and SMART, thus demonstrating the tremendous expansion of members in this protein superfamily. Structural studies showed that the JmjC domain has a very characteristic topology. It forms a double-stranded β-helix (DSBH) fold, also known as jelly-roll motif, that normally consists of eight antiparallel β-strands [6]. This DSBH topology is characteristic for the cupin metalloenzymes [7] and for the JmjC domain containing proteins [3]. Cupin metalloenzymes constitute a large and diverse superfamily of proteins with enzymatically active and non-active members. They contain at least one DSBH or jelly-roll motive. Secondary structure elements that surround the DSBH are used further to define different subfamilies (for review see [6]). The DSBH fold in JmjC domain containing proteins can form an enzymatically active pocket by coordinating di-iron (Fe(II)) and the co-substrate 2-oxoglutarate (2OG). In almost all cases, the two-electron oxidation of the "prime" substrate is coupled to the conversion of 2OG into succinate and CO_2 _[8]. Therefore, this class of enzymes is also known as nonheme-Fe(II)-2-oxoglutarate-dependent dioxygenases.

Oxidative reactions catalysed by 2OG-dependent dioxygenase are critical steps in the biosynthesis of metabolites, or post translational modification of interacting target proteins involving hydroxylation or demethylation and DNA/RNA repair of N-methylated nucleic acids [6,9]. As such, 2OG-dependent dioxygenases are of immanent biological importance. A number of JmjC domain containing proteins are involved in regulating key developmental processes during mammalian embryogenesis by acting as transcription factors. An example of this subgroup of JmjC domain containing proteins is jumonji itself (now called Jarid2), which can act as a transcriptional repressor. Jarid2 inhibits cyclin D1 transcription during heart and brain development and thereby controls cellular proliferation and differentiation in these organs during morphogenesis [10-12]. Other examples of JmjC-domain proteins that function as transcriptional repressors are the retinoblastoma-binding protein 2 (Jarid1a, also known as RBP2), which is required for the differentiation of various cell types including muscle, bone and myeloid cells [13] and hairless, a nuclear co-repressor of multiple nuclear hormone receptors such as the vitamin D receptor, the thyroid hormone receptor and the retinoic acid-receptor-related orphan receptor α [14-16]. Mutations in hairless result in humans and mice in alopecia, a form of hereditary hair loss due to defects in the regeneration of hair follicle cells [17-19]. More recently, JmjC-domain containing proteins have gained emerging importance in histone modification and chromatin regulation. The JmjC domain containing histone demethylases (JHDMs) can catalyse lysine demethylation of histones through an oxidative reaction that requires iron Fe(II) and 2OG. Histone methylation marks have important roles in regulating gene expression and are central to the control of epigenetic information that regulates cell fate and identity [20]. Until now, JHDMs have been reported to be capable to reverse H3K36 (JHDM1/FBXL11, [21], JMJD2A, [22,23], Rph1, [24,25], Jhd2, [25], Gis1, [25], Jhd1, [25]), H3K9 (JHDM2A/JMJD1A, [26]), and both H3K9 and H3K36 methylation (JHDM3A to JHDM3D/JMJD2A to JMJD2D, [23,27-30]). In addition, the JARID subfamily of JmjC-domain containing proteins have been shown to possess H3K4 demethylase activity in different species (JARID1A/RBP2, [31,32], JARID1C/SMCX, [31,33], JARID1D/SMCY, [34], JARID1B/PLU-1/Yjr119Cp/Jhdp2, [31,35,36], Lid, [26,37-39], and JMJ2 [40]). Most recently, the JmjC-domain containing proteins UTX and JMJD3 have been shown to function as H3K27 specific demethylases and demonstrated to play an important role in animal body patterning and regulation of inflammatory responses [41-44]. Through this activity the JHDMs have been postulated to regulate diverse cellular processes including control of cell proliferation, heterochromatin assembly and gene expression [26,28,30,34,45,46]. However, for most of the newly discovered JHDMs gene ablation studies *in vivo *have as yet not been carried out leaving the possibility open that in addition to histones other substrates might be targets of these proteins.

Many of the different classes of JmjC domain containing proteins possess complex domain architectures by having at least one additional protein domain besides the JmjC domain. Here, the JHDMs and Jarid proteins are good examples. In addition to the JmjC domain they have PHD, Tudor, Bright/Arid and zinc finger domains, which have recently been shown to interact with methylated histone tails and are required for their catalytic function [33,38,47]. In contrast, other members in this protein superfamily have no other recognisable protein domain apart from the JmjC domain. Important members of this subgroup of JmjC domain only proteins are the 2OG-dependent dioxygenase factor inhibiting HIF (Hif1an, also known as FIH) and the PHD hydroxylases, which are involved in the maintenance of oxygen homeostasis. Hif1an was the first structurally resolved mammalian JmjC domain containing protein [48,49] and was shown to function as an asparagine hydroxylase for the hypoxia inducible factor α (HIF1α), a transcription factor that is central to oxygen homeostasis in both physiological and pathophysiological processes (for review see [50]). The PHD hydroxylases PHD1 to PHD3 also target HIF1α by hydroxylating proline residues. Under normal oxygen conditions the hydroxylation reactions carried out by PHDs induce the proteasomal degradation of the HIF1α subunit, whereas under hypoxia HIF1α is not hydroxylated and translocates from the cytoplasm to the nucleus where it activates transcription of genes needed for adaptation to low oxygen levels. AlkB is another member of the JmjC domain only proteins. As a 2OG-dependent oxygenase AlkB can repair DNA that is methylated at 1-methyladenine and 3-methylcytosine by oxidation of the methyl group [51,52]. AlkB and related proteins define a subfamily of small 2OG-dependent oxygenases that are involved in DNA and RNA repair [53].

In the past, we have analysed the function of the *phosphatidylserine receptor *(*Ptdsr *or also known as *PSR *or *PtdSerR*), a gene that also encodes a JmjC domain only protein [54,55]. First described as a cell surface receptor for the recognition and engulfment of apoptotic cells [56], recent studies have raised serious doubts about its involvement in apoptotic cell clearance. Five groups independently reported a nuclear localisation of Ptdsr, which clearly contradicts the hypothesis that the protein might function as a transmembrane receptor [57-61]. Furthermore, a comprehensive analysis carried out by us in *Ptdsr*-knockout mice demonstrated no impairment in the removal of apoptotic cells in *Ptdsr*-deficient embryos and macrophages ([54] for *Ptdsr*^*tm*1*Gbf*^) thereby contradicting two previous studies that reported defects in apoptotic cell clearance in two other *Ptdsr*-knockout mice ([62] for *Ptdsr*^*tm*1*Flv*^, and [63] for *Ptdsr*^*tm*1*Ysfk*^). All studies involving *Ptdsr*-knockout mice conclusively demonstrated that the gene is essential for normal development. *Ptdsr*-deficient mice are dying at birth and display severe differentiation defects in many tissues such as brain, eyes, lung, kidney, liver and intestine at different stages of embryogenesis [54,62,63]. Furthermore, *Ptdsr *plays an important role during heart development and ablation of *Ptdsr *function is associated with complex cardio-pulmonary malformations that resemble the human congenital heart syndrome Tetralogy of Fallot [55].

Although *Ptdsr *is clearly an important differentiation-promoting gene during embryogenesis its molecular function is still unknown. Based on a bioinformatic analysis of the cloned *Hydra *homologue *PSR*, Cikala and colleagues were the first to suggest that the protein might function as a 2OG-dependent dioxygenase that is capable of modifying unknown nuclear targets [57]. In recognition of the still unknown function of the "phosphatidylserine receptor gene" the International Committee for Standardized Genetic Nomenclature in Mice (ICSGNM) has most recently revised its name. Ptdsr has been renamed into *jumonji domain containing gene 6 *(*Jmjd6*). We will obey the guidelines of the ICSGNM and use this name and the gene symbol *Jmjd6 *throughout the remainder of this article.

To gain more insights into possible Jmjd6 functions using sequence homology approaches, we developed a semiautomated process to extract all available Jmjd6 homologous protein and nucleic acid sequences from public databases. This comprehensive data set was used to identify proteins with sequence homology to Jmjd6 in 62 species across all major living phyla. In addition, this allowed us to identify new *Jmjd6/JMJD6 *splice variants in mice and humans and to analyse the genomic neighbourhood of the gene in vertebrates. Immediately downstream of *Jmjd6 *we found the gene *1110005A03Rik*, which is predicted to encode a putative methyltransferase, which is as yet not further biochemically characterised. Interestingly, we found *1110005A03Rik *to be affected by deletions in two previously reported *Jmjd6 *knockout mice. To predict possible structure-function relationships of Jmjd6, we employed a comparative modelling approach of the three-dimensional structure of the protein and annotated probable functional residues based on sequence conservation. To integrate Jmjd6 into the superfamily of JmjC-domain containing proteins, we assembled a non-redundant set of 313 DSBH-domain containing proteins by using our semiautomatic retrieval script and grouped these according to phylogenetic relationships. Taken together, our results further support a conserved enzymatic function of Jmjd6 most likely as a classical non-heme, Fe(II)- and 2OG-dependent dioxygenase. It also shows that Jmjd6 is closely related to Jmjd4 and JFP6, and constitutes together with these two proteins a subgroup of JmjC domain containing proteins. Moreover, we identified in protista, fungi and plants proteins with sequence homology to Jmjd6 that diverge from the classical "JmjC-only domain" structure. Some of these proteins have in comparison to the animalia Jmjd6 extended C-terminal regions that contain additional domains that might have been lost during evolution.

With these approaches we establish here an overall framework of information that will facilitate not only further research regarding the functional characterisation of Jmjd6 but also of other members within the DSBH-domain containing superfamily of proteins.

## Results

### Retrieval and analysis of a comprehensive set of Jmjd6 orthologs and other JmjC domain containing proteins

**Figure 1:**
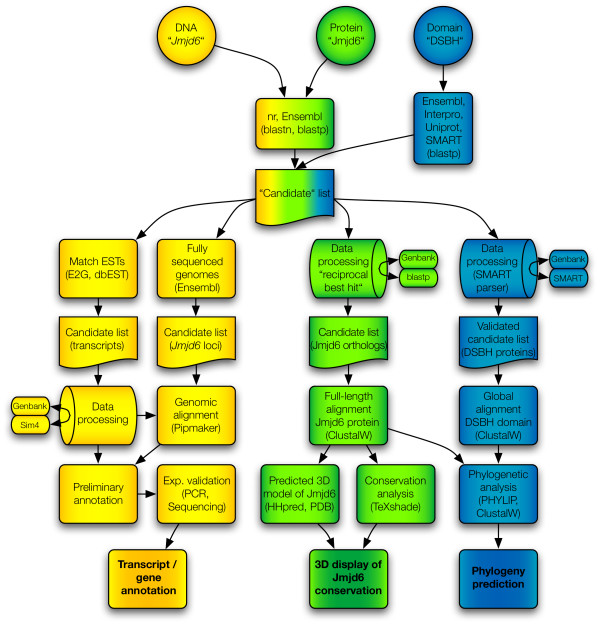
**Computational pipeline flow used for the retrieval and analysis of DSBH/JmjC-domain containing proteins**. It contains three parallel procedures. Indicated in yellow colour are searches and analysis approaches based on DNA sequences, data processing approaches based on protein sequences are coloured green, and phylogenomic analysis methods based on DSBH protein domains are shown in blue. Initial queries are depicted as circles, databases as ovals, candidate gene and protein lists as cut-off boxes, and methods/analysis tools as normal boxes, respectively. Barrels indicate Perl script based analysis of retrieved results. Candidate lists of the individual workflows are shown and the final results of the analysis are depicted in bold. Arrows indicate the flow of information and the connections between different search routines and methods. Sources of queried databases and the analysis software used are given in the respective box elements.

We developed a semiautomatic process to extract all available Jmjd6 homologous protein and nucleic acid sequences from public databases. The computational pipeline consists of three parallel procedures based on "DNA", "protein", and "protein domain" searches (Figure 1). With this approach, we generated a candidate list of 11.376 sequence entries including all putative *Jmjd6 *orthologs and all JmjC/DSBH domain containing proteins present in Genbank, Ensembl, Uniprot, PFAM, Interpro, and SMART, independent of their annotation. The goal of our semiautomated computational pipeline was then to identify and systematically analyse all orthologous *Jmjd6 *loci, all transcripts encoded by the murine and human *Jmjd6/JMJD6 *loci, the degree and distribution of sequence conservations in Jmjd6 proteins, and their phylogenetic relationship to other JmjC/DSBH domain containing proteins. In the end, the combination of these analysis approaches enabled us to identify 62 proteins with significant sequence homology to Jmjd6 and to establish a set of 313 non-redundant DSBH domain containing proteins in human and eight model organisms.

We started the "DNA" sequence based retrieval and analysis approach (depicted in yellow in Figure 1) by identification of all mouse *Jmjd6 *homologous sequences available in Ensembl (release 44). This candidate loci list contained homologous *Jmjd6 *loci in fully annotated genomes as well as *Jmjd6 *sequence fragments from ongoing sequencing projects. To identify conserved coding and non-coding sequences within these genomic regions we undertook a PipMaker analysis of retrieved sequences that showed a similar genomic organisation in comparison to the murine *Jmjd6 *locus. Next, we mapped all available murine and human ESTs in dbEST to the murine and human *Jmjd6*/*JMJD6 *loci, respectively, using the program e2g. Extensive validations of each EST employing Sim4 with our own perlscript for data retrieval, handling, examination and sorting of the results led to a comprehensive analysis of the murine and human *Jmjd6*/*JMJD6 *loci and a detailed annotation of all encoded transcripts.

To identify all *Jmjd6 *orthologs, we employed a blastp search against Ensembl and nr databases using a unique N-terminal sequence of the murine Jmjd6 protein as a template ("protein" based search and analysis, depicted in Figure 1 in green). The candidate matches were individually verified in a *Mus musculus *restricted blastp database search using the reciprocal best BLAST hit criterion and a perlscript for automatic data processing. These led to the identification of Jmjd6 proteins in other species that are closely related to and might be orthologs of the murine Jmjd6 protein. A full length ClustalW alignment of proteins with significant homology to Jmjd6 was processed using a TeXshade based conservation analysis and also as input for a 3D structure prediction of the catalytic core domain. By combining these results, we developed a novel way of predicting residues that are most likely essential for the catalytic activity of the protein based on their degree of conservation.

To analyse the phylogenetic evolution of the Jmjd6 JmjC domain, we developed SMART-parser. SMART and SMART-parser were used to analyse the domain composition and extract the sequence information from a set of sequences that was derived from the Ensembl, Interpro, Uniprot and SMART databases ("DSBH-domain" routine, depicted in Figure 1 in blue). This set comprises all JmjC-, Pfam-JmjC, Blast-JmjC-, P4Hc-, Pfam-2OG-FeII-Oxy- and Pfam-PhyH-domain containing sequences from human, mouse, zebrafish, pufferfish, nematode, fruit fly, and yeast. These different domain names have been used in the past to annotate the JmjC domain in DSBH fold containing proteins. The SMART-parser verified sequences were used as an input for a global ClustalW alignment. A PHYLIP processing of all ClustalW alignments allowed the analysis of the Jmjd6 phylogeny in relationship to all other identified DSBH domain containing proteins.

### Comparative analysis of *Jmjd6 *homologous loci in vertebrates

**Figure 2:**
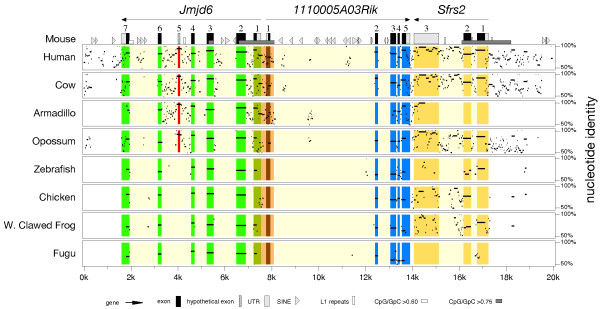
**Percent identity plot of the chromosomal regions encompassing the *Jmjd6 *locus in vertebrate species**. The genomic regions of eight vertebrate species identified as putative *Jmjd6 *orthologous loci were aligned with the mouse *Jmjd6 *locus and its neighbouring genes using PipMaker. Green, blue and yellow overlays highlight the conservation of the annotated exons/coding sequences of the individual genes found in the reference sequence. The orange overlay indicates conserved sequences of the first exons of the *Jmjd6 *and *1110005A03Rik *genes and sequences outside of the coding elements. The red overlay marks a high-scoring segment in all analysed mammalian species supporting the presence of at least one additional exon (No. 5) that is not included in the current Ensembl gene annotation (build 44). Transcriptional orientations of the genes, their exons, UTRs, repetitive elements, and CpG islands are in the symbol key at the figure bottom.

In the mouse, *Jmjd6 *is localised in a gene dense region on distal chromosome 11. Using different gene targeting strategies three loss-of-function alleles of *Jmjd6 *have been generated that display conflicting phenotypes [54,62,63]. To analyse the immediate genomic neighbourhood of *Jmjd6 *and to identify all transcripts encoded by the locus, we undertook a comparative sequence analysis of this gene region in different species. In Ensembl, the mouse *Jmjd6 *gene is annotated to consist of six exons compromising 6 kb (Ensembl release 44). Immediately downstream of *Jmjd6 *two genes are located, *1110005A03Rik*, an expressed sequence tag (EST) encoding a putative arginine protein methyltransferase [64], and *Sfrs2*, encoding the splicing factor SC 35 [65]. To analyse the genomic region encompassing *Jmjd6 *and *1110005A03Rik *in more detail, we performed a comprehensive database screen. First, we searched all fully sequenced genomes present in Ensembl build release 44 for an annotated *Jmjd6 *gene. We identified *Jmjd6 *homologous genes or genomic *Jmjd6 *fragments in 17 mammalian, 1 avian, 1 amphibian, 5 fish, 2 urochordata, and 4 invertebrate genomes (Table 1). The identified putative *Jmjd6 *orthologous loci were then searched for the presence of the *1110005A03Rik *gene. In 22 out of the 30 analysed genomes we found both genes located close together. Remarkably, in all analysed invertebrate species *Jmjd6 *and *1110005A03Rik *were not found to co-localise despite the presence of both genes at other chromosomal locations in the examined genomes. Next, we extracted 50 to 100 kb of genomic sequence encompassing the *Jmjd6 *locus and adjacent loci of these 22 species and used these for an initial PipMaker sequence analysis (data not shown). To carry this out, the mouse sequence was first analysed for repetitive sequence elements using RepeatMasker and secondly, the positions of all coding sequences/exons in *Jmjd6*, *1110005A03Rik*, and *Sfrs2 *were identified and annotated using the BLAST algorithm. The masked and annotated mouse sequence was then finally used for interspecies pairwise alignment with the human, bovine, armadillo, opossum, zebrafish, chicken, western clawed frog, and puffer fish sequences using the program PipMaker [66]. These species were selected for the analysis either based on their large phylogenetic distance to the mouse or on the availability of high quality, full-coverage sequence information in the extracted regions. The resulting alignments of similar regions in the genomic sequences were summarised as "percent identity plot" (pip) displaying sequence identity in combination with the annotated repeats and transcriptional units (Figure 2). The pip shows a high degree of sequence conservation in the coding regions of the three genes and confirms the accuracy of the initial exon assignments annotated by Ensembl. However, a detailed inspection of the *Jmjd6 *locus identified further high-level conserved sequences (= 90%) in between the annotated exons 4 and 5 (red overlay in Figure 2). This sequence conservation suggested the presence of an up to now overseen additional exon, at least in the mammalian species. In addition, the pip confirms the close association of the *Jmjd6 *and *1110005A03Rik *genes in all analysed organisms. *Jmjd6 *and *1110005A03Rik *are arranged in a head-to-head transcriptional orientation with a short intergenic distance of 65 bp between both genes. This suggests that these two genes build a conserved transcriptional unit that possibly shares a bi-directional promoter region. This is further supported by the presence of a conserved short non-coding sequence in between the translational start sites of these two genes (orange overlay in Figure 2). Interestingly, we observed not only a conserved association of the *Jmjd6 *and *1110005A03Rik *genes in this genomic region, but also of *Sfrs2*, which is present in this region in all species with the exception of the puffer fish (Figure 2). In the mouse, *Sfrs2 *is located 6.3 kb distal of *Jmjd6 *and 170 bp distal to *1110005A03Rik *(Figure 2).

**Table 1:** Species distribution and chromosomal locations of *Jmjd6 *orthologs in mammalian and non-mammalian genomes

**Species name**	**Common name**	**Genomic location**	**Cluster with *1110005A03Rik***	**Cluster with *Sfrs2***
*Homo sapiens*	Human	17	**+**	**+**
*Pan troglodytes*	Chimp	17	**+**	**+**
*Macaca mulatta*	Macaque	16	**+**	**+**
*Mus musculus*	Mouse	11	**+**	**+**
*Rattus norvegicus*	Rat	10	**+**	**+**
*Cavia porcellus*	Guniea pig	Gene scaffold 591	**+**	
*Spermophilus tridecemlineatus*	Ground squirrel	Scaffold 5110	**+**	
*Tupaia belangeri*	Tree shrew	Scaffold 5346	**+**	
*Canis familiaris*	Dog	18	**+**	**+**
*Felis catus*	Cat	Scaffold 295	**+**	
*Bos taurus*	Cow	19	**+**	**+**
*Myotis lucifugus*	Microbat	Scaffold 462	**+**	
*Erinaceus europaeus*	Hedgehoc	Scaffold 670	**+**	**+**
*Dasypus novemcinctus*	Armadillo	Scaffold 584	**+**	
*Echinops telfairi*	Tenrec	Scaffold 620	**+**	
*Monodelphis domestica*	Opossum	2	**+**	**+**
*Ornithorhynchus anatinus*	Platypus	Ultra 430	**+**	
*Gallus gallus*	Chicken	18	**+**	
*Xenopus tropicalis*	Western clawed frog	Scaffold 178	**+**	**+**
*Gasterosteus aculeatus*	Stickleback	Group XI	**+**	
*Oryzias latipes*	Medaka	Scaffold 1014		
*Tetraodon nigroviridis*	Tetraodon	SCAF 13844	**+**	
*Takifugu rubripes*	Fugu	Scaffold 519	**+**	
*Danio rerio*	Zebrafish	3	**+**	**+**
*Ciona intestinalis*	Sea squirt	3p		
*Ciona savignyi*	Sea squirt	Reftig 1		
*Drosophila melanogaster*	Fruitfly	3R		
*Aedes aegypti*	Aedes	Super contig 1.887		
*Anopheles gambiae*	Mosquito	X		
*Caenorhabditis elegans*	C. elegans	IV		

Chromosomal locations have been taken from ENSEMBL, + indicates the presences of *1110005A03Rik *or *Sfrs2 *in the immediate genomic neighbourhood of *Jmjd6*.

**Figure 3:**
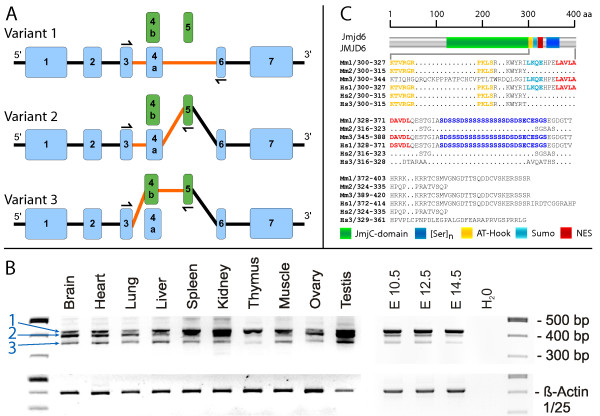
**Identification and characterisation of new splice variants of *Jmjd6***. ***(A) ***Two additional putative exons (green boxes) were identified in addition to the transcript annotated in Ensembl (variant 1) by screening all public EST databases. Alternative splicing using these two exons results in the generation of two additional transcripts of *Jmjd6 *(variants 2 and 3, respectively). Half arrows and orange lines in the schematic presentation of the transcripts highlight the combination of exons detected by RT-PCR. Numbering of *Jmjd6 *exons includes the additional identified exon variants 4b and 5. ***(B) ***Expression analysis of alternative *Jmjd6 *transcripts in adult mouse organs and embryonic stages (E) using RT-PCR confirmed the presence of all three transcripts at different mRNA expression levels in the analysed samples. Half arrows in (A) indicate the position of the PCR primers used for the amplification. Blue numbers and arrows in (B) point to PCR fragments amplified from splice variants 1 to 3. Amplification of the housekeeping *β-actin *gene was used as a loading control. ***(C) ***The effects of the different splicing events on the C-termini are shown in the gapped alignment from amino acid 300 onwards. The full length human JMJD6 and murine Jmjd6 proteins are represented in the upper part of the figure. Nearly all novel splice variants identified in the mouse (Mm 2) and in humans (Hs 2–3) are truncated at the C-terminus in comparison to splice variant 1 (Mm 1 and Hs 1) and are predicted to contain no poly-serine stretch ([Ser]_n_). In contrast, the JmjC domain is not affected (green box). Predicted AT-hook domain, sumoylation recognition site (Sumo), and nuclear export signals (NES) are annotated as depicted at the bottom.

To further test the hypothesis that an additional exon in *Jmjd6 *might generate alternative transcripts of this gene, we investigated all available mouse *Jmjd6 *EST data currently present in dbEST. Using e2g, a web-based server that allows an efficient mapping of large EST and cDNA datasets to genomic regions [67], all *Jmjd6 *ESTs were mapped to the mouse locus and adjacent regions. The resulting EST hits represent the entire putative *Jmjd6 *coding region including the transcribed and untranslated regions (UTRs). These cDNA sequences were then extracted and individually aligned with the mouse *Jmjd6 *genomic sequence using Sim4, a computer program that allows an efficient assignment of cDNAs to large genomic regions by accounting for the position of introns [68]. The Sim4 validated EST sequences were then manually curated and all predicted *Jmjd6 *transcripts were assembled and annotated to the genomic locus. As a result of this analysis, we identified a new variant of exon 4 in *Jmjd6 *and found an additional, before overseen exon in the conserved region between exons 4 and 5. This allowed us, solely based on the EST data, to predict the presence of two novel *Jmjd6 *splice variants in addition to the main transcript of 1.4 kb currently annotated in Ensembl (Figure 3A). To validate *in vivo *the existence of the predicted splice variants, we performed RT-PCR expression analysis. Various major organs from adult mice as well as whole embryos at different developmental stages (E) were used for the analysis. RT-PCR amplification yielded three DNA fragments indicative for the predicted transcripts in all analysed samples at varying levels (Figure 3B). Taken together, this confirmed the computational prediction of two new *Jmjd6 *splice variants encoded by the mouse locus and demonstrated that *Jmjd6 *consists of seven exons, with exon 4 occurring in two different variants 4a and 4b, respectively. At the protein level both new detected splice variants (transcripts 2 and 3 in Figure 3A) affect the C-terminal part of the Jmjd6 protein. In comparison to the main transcript annotated in Ensembl (transcript 1 in Figure 3A), either a stretch of 9 amino acids starting at position 302 is exchanged against 31 other residues (transcript Mm3 in Figure 3C) or a premature stop codon is introduced resulting in a truncated protein of 314 amino acids (transcript Mm2 in Figure 3C). Using the CBS-prediction service, we identified in this region of the protein a putative sumoylation site (probability score 92%) and a nuclear export signal (probability score 96%) using NetNES. Furthermore, this region contains the previously described putative AT-hook domain [57] and the poly-serine stretch domain ([69,70] Figure 3C). All these potential interaction sites are not present in the protein encoded by transcript Mm2. Transcript Mm3 differs in comparison with the main transcript (Mm1) in a stretch of 31 amino replacing the putative AT-hook containing region (Figure 3C).

Based on the identification of the highly conserved sequence coding for the new exon 5 in all mammalian species (Figure 2 and data not shown), we performed a similar e2g and Sim4 analysis using the human *JMJD6 *locus and human EST database entries. This analysis confirmed the presence of exon 5 containing *JMJD6 *transcripts in humans and also the presence of two different variants of exon 4 in the human genome. Using again RT-PCR, we validated the expression of these transcripts *in vivo*. Similar to the mouse we found expression of the three transcripts in 10 human tissues [see Additional file 1]. These, as well as the mouse alternative transcripts, were further validated and confirmed by sequencing (Accession numbers EF527404 for Mm2 and EF527405 for Mm3 for mouse *Jmjd6 *and EF527406 for Hs2 and EF527407 for Hs3 for human *JMJD6 *transcript variants, respectively). In comparison to the new identified mouse transcript variants, both human transcript variants are predicted to generate truncated proteins. Neither of these predicted proteins appear to encode a nuclear export signal, sumoylation site, AT hook domain or poly-serine stretch at the C-terminal part of the protein (Fig. 3C). As an insertion of premature translation termination codons might result in nonsense-mediated decay (NMD) of the corresponding mRNA [71], we analysed if the mouse transcript variant 2 and human splicing variants 2 and 3 might be subjected to degradation by the NMD pathway. Using RT-PCR assays for each of these variants we found that this is not the case. Primers located in the new introduced exons and at the 3'-end of each transcript yielded products of the expected size, indicating that these variants are indeed expressed and most likely functional [see Additional file 2]. The human and mouse splice variants seem to be expressed at different mRNA levels depending on the tissue analyzed (see Figure 3 and [see Additional file 2]), with mouse splice variant Mm1 expressed in higher amounts in the developing embryo (Figure 3). Interestingly, an e2g and Sim4 analysis performed in non-mammalian species did not result in the recognition of mouse and human related *Jmjd6 *splice variants in these organisms (data not shown).

**Figure 4:**
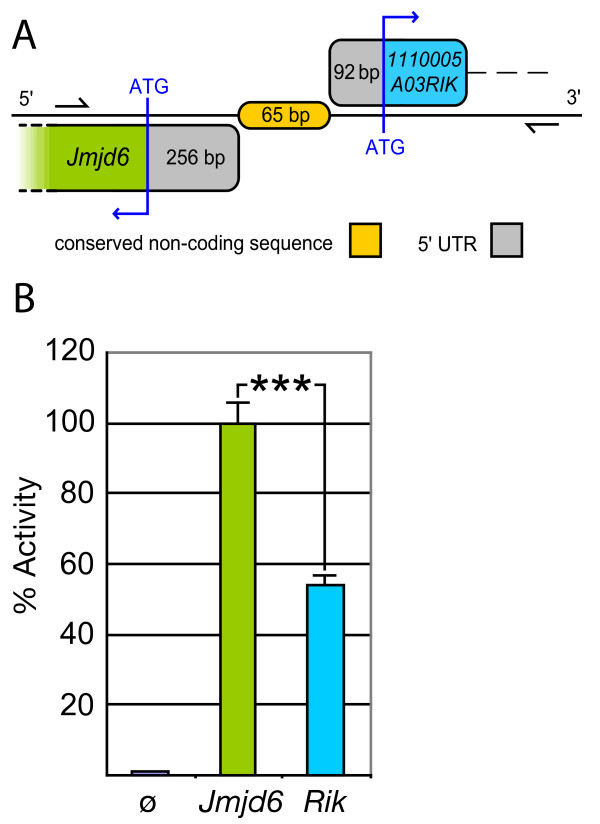
**Identification of the *Jmjd6 *– *1110005A03Rik *bi-directional transcriptional unit**. ***(A) ***Schematic overview of the 65 bp non-coding and non-overlapping intergenic region between the murine *Jmjd6 *and *1110005A03Rik *(*Rik*) genes. The conserved sequence element identified using PipMaker in Figure 2 is shown in orange. 5'-untranslated regions (UTRs) are indicated in grey, and first exon coding sequences of the *Jmjd6 *gene in green and of the *1110005A03Rik *gene in blue, respectively. The region indicated by two half arrows was used for promoter activity assays presented in (B). It was amplified and cloned to yield an 840 bp test fragment ***(B) ***This putative bi-directional promoter fragment was used in a luciferase reporter gene assay to measure transcriptional activity. Basal promoter activity was found for both transcriptional orientations compared to a promoterless control (pGL3) vector. The 5'-3' orientation of the fragment (*1110005A03Rik *orientation, *Rik*) yielded only half of the transcriptional activity as it was measured for the 3'-5' orientation (*Jmjd6 *orientation, *Jmjd6*). The values reported for the transfection experiments are means ± standard deviation of three independent triplicate experiments. P value was determined using Student's t-test. *** P < 0.001.

To analyse the conserved, putative bi-directional promoter element in between *Jmjd6 *and *1110005A03Rik*, we tried to identify potential regulatory motifs in the genomic sequence encompassing the intergenic region, the 5'-UTRs and the first exons of both genes. We isolated a 840 bp fragment covering the genomic region between *Jmjd6 *and *1110005A03Rik *(Figure 4A) that contained several predicted transcription factor binding sites based on a MatInspector analysis (data not shown). This fragment was further analysed using a promoter-less luciferase reporter gene assay. In comparison to controls, the selected genomic fragment clearly possesses transcriptional promoter activity when transfected into primary mouse embryonic fibroblast cells (MEFs) and various cell lines (Figure 4B and data not shown). Moreover, this transcription activating activity was found to be orientation dependent. In the forward strand orientation (which controls *in vivo 1110005A03Rik *expression according to the genomic annotation in Ensembl) the fragment showed only half of the activity as compared to the reverse strand orientation (Figure 4B), which controls expression of *Jmjd6 in vivo*. This demonstrated that the short intergenic region between *Jmjd6 *and *1110005A03Rik *is capable to control basal expression of both genes, although this bi-directional transcriptional unit seems to activate both genes at different levels.

We then went on to investigate the consequences on *Jmjd6*, *1110005A03Rik *and *Sfrs2 *expression of the different gene targeting strategies used to generate the three *Jmjd6 *mouse knockout alleles. In all three *Jmjd6 *mutant mice (*Jmjd6*^*tm*1*Flv*^, generated by the Flavell laboratory; *Jmjd6*^*tm*1*Ysfk*^, generated by the Fukui laboratory; and *Jmjd6*^*tm*1*Gbf*^, generated in our laboratory) the first two or three exons of the gene have been deleted. However, an alignment of restriction enzyme sites used to construct the different gene targeting vectors reveals that in *Jmjd6*^*tm*1*Flv *^and *Jmjd6*^*tm*1*Ysfk *^mice, in contrast to the *Jmjd6*^*tm*1*Gbf *^mouse line, exon 1 of *1110005A03Rik *was deleted in addition [see Figure A in Additional file 3]. This was confirmed when we analysed the expression of all three genes in homozygous embryos harvested from the three mutant mouse lines. *Jmjd6*^*tm*1*Flv*-/- ^and *Jmjd6*^*tm*1*Ysfk*-/- ^animals show no expression of *1110005A03Rik *and *Jmjd6*, while the expression of *Sfrs2 *remains unaffected by the gene targeting. In *Jmjd6*^*tm*1*Gbf *^homozygous embryos *1110005A03Rik*, and *Sfrs2 *are expressed comparable to wild type levels [see Figure B in Additional file 3] and as previously shown [54], only the expression of *Jmjd6 *is ablated in this mouse mutant line. This was further supported by semiquantitative expression analysis of *1110005A03Rik *in *Jmjd6*^*tm*1*Gbf*-/- ^embryos and primary fetal liver-derived macrophage cultures. RT-PCR analysis of serial diluted cDNA shows equal expression of *1110005A03Rik *in *Jmjd6*^*tm*1*Gbf *^animals in comparison to wildtype controls in all analysed samples [see Figure C in Additional file 3]. Thus, the *Jmjd6*^*tm*1*Flv *^and *Jmjd6*^*tm*1*Ysfk*^alleles represent double knockouts of the *Jmjd6 *and *1110005A03Rik *genes, while the *Jmjd6*^*tm*1*Gbf *^allele ablates only *Jmjd6 *gene expression.

### Phylogenetic analysis of the Jmjd6 protein

**Figure 5:**
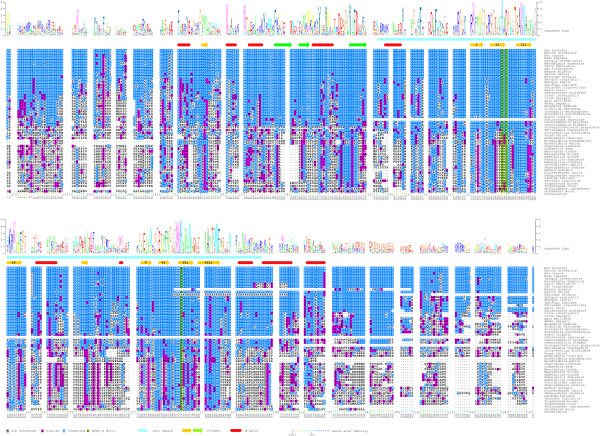
**Multiple sequence alignment of homologous Jmjd6 proteins**. The sequences of 54 homologous Jmjd6 proteins were used for the calculation of a multiple sequence alignment (ClustalW). The numbering of the residues is according to the mouse protein sequence. Identical residues were shaded in blue and similar residues in magenta. Green shading indicates the predicted catalytic residues of the HxDx_n_H facial. Sequence insertions in individual species outside of the conserved core regions of the mouse Jmjd6 protein were masked out and the maximum numbers of amino acids in these insertions are indicated [n]. Based on the sequence conservation, a frequency corrected sequence logo was calculated (shown above the alignment) and the mouse sequence conservation was highlighted in colour dots according to their degree of conservation (shown as colour code in percent identity below the alignment). The JmjC domain (light blue bar) and additional identified secondary structure elements (red cylinders: α-helices; yellow and green arrows: β-strands) were annotated to the sequence as indicated at the bottom and the β-strands forming the jelly-roll of the JmjC domain were numbered (I-VIII). Green arrows represent the β-sheet that extends the major sheet outside of the jelly-roll.

Following the identification of Jmjd6 orthologues in 30 of the 34 species annotated in Ensembl (Table 1), which implied a broad distribution of *Jmjd6 *during evolution, we continued to search for additional homologous proteins in other species. To identify further candidate proteins, we performed a comprehensive database (nr) search using the blastp algorithm and the N-terminal part encompassing amino acids 53 to 152 of the mouse Jmjd6 protein. For the search strategy, we explicitly excluded the highly conserved JmjC domain to reduce the number of false positive hits representing Jmjd6-unrelated JmjC domain containing proteins. To ascertain that we select for the subsequent analysis in each species the sequence with the highest homology to Jmjd6, we used the "reciprocal best BLAST hit" criterion [72]. The obtained full-length database entries were used for a *Mus musculus*-restricted blastp-search of the nr database and for each species only those sequences matching Jmjd6 as the best BLAST hit criterion were retained for the analysis. As some sequences are only available from early stage annotation projects (low-sequence coverage genomes, [73]), we selected 54 out of 62 sequences due to quality measures as a working package for the calculation of a multiple sequence alignment by employing ClustalW. Following five iterations of re-alignment of the protein sequences the multiple sequence alignment was shaded with respect to amino acid identity/similarity and a protein sequence logo was generated and adjusted to the background frequency of each amino acid in the alignment. In addition, predicted secondary structure elements and SMART domains of the mouse Jmjd6 protein were added (Figure 5). The retrieved reciprocal best BLAST hits from the different species and the quality of the multiple sequence alignment allowed us to confirm the high homology of these 54 proteins to the murine Jmjd6 protein (Figure 5). Many of those are with no doubt true orthologous proteins of the mouse Jmjd6. These are found in the mammalia, aves, amphibia, actinopterygii, urchordata, echinodermata, insecta, nematoda, platyhelminthes, and cnideria and demonstrate the remarkable conservation of the *Jmjd6 *gene during evolution (Figure 5 and [see Additional file 4]) as previously also noted by others ([56,57]). However, some proteins identified in plants, protista, fungi differ from the "JmjC only" domain structure characteristic for the Jmjd6 proteins of animalia. For example, some of them contain nearly five times more amino acids or have in addition to the JmjC-domain single F-box domains [see Additional files 4 and 9]. Nevertheless, they are the closest relatives to the mouse Jmjd6 as defined by the best reciprocal BLAST hit criterion. Thus, it is difficult to decide if these proteins might be true orthologs of the animalia Jmjd6 proteins. If they are, it could mean that additional protein domains have been lost from an ancient Jmjd6 protein during evolution in the animalia lineage.

To explore potentially important residues for Jmjd6 function we compared the degree of sequence conservation across the aligned sequences and included in the alignment predicted tertiary structure elements of the protein. First, our analysis showed the presence of a 2-His-1-carboxylate facial triad (HxDx_n_H), as a common structural motif among non-heme iron(II) enzymes, present in all identified Jmjd6 proteins (Figure 5), suggesting an evolutionary conserved enzymatic function of Jmjd6. We could identify within the predicted JmjC domain eight β-strands (I-VIII) that have been shown to form the characteristic jelly-roll topology in other structurally resolved JmjC domain containing proteins [6]. The amino acids encompassing these β-strands are highly conserved in all proteins. 50% of all β-strands forming amino acids are identical in at least 85% of the analysed species suggesting the spatial and functional conservation of the JmjC-domain in all analysed proteins. The only exception we found is in between β-strands IV and V. At this position we found an insertion of sequences of variable amino acid conservation among species and of varying length as it has been previously described for other JmjC domain containing proteins [6,22,53]. Second, within the C-terminal region of the Jmjd6 protein preceding the last β-strands of the recognised jelly-roll the degree of sequence conservation is decreased. In contrast to the metazoan species showing a high degree of sequence identity, all other species display a significantly reduced similarity in this region. Furthermore, only the metazoan species have a poly-serine stretch in the C-terminal region of the Jmjd6 protein (Figure 5 and [70]). We could not identify any comparable sequence motif in the other species but looking for chemical properties of amino acids present at this position in proteins of non-metazoan species we found an enrichment of polar amino acids in this part of the protein (data not shown). This suggests at least a conservation and fortification of physico-chemical properties in this region during evolution. Third, the part N-terminal of the JmjC domain shows in comparison to the C-terminus a higher degree of conservation. We found that 20% of the amino acids 59–140 are identical in at least 90% of the analysed proteins (Figure 5). In addition, we identified one highly conserved and three less conserved β-strands in this region. These findings suggest the presence of an important structural element of the Jmjd6 protein in close vicinity to the predicted JmjC domain (see below).

### Comparative modelling and analysis of the conserved putative catalytic core domain of Jmjd6

**Figure 6:**
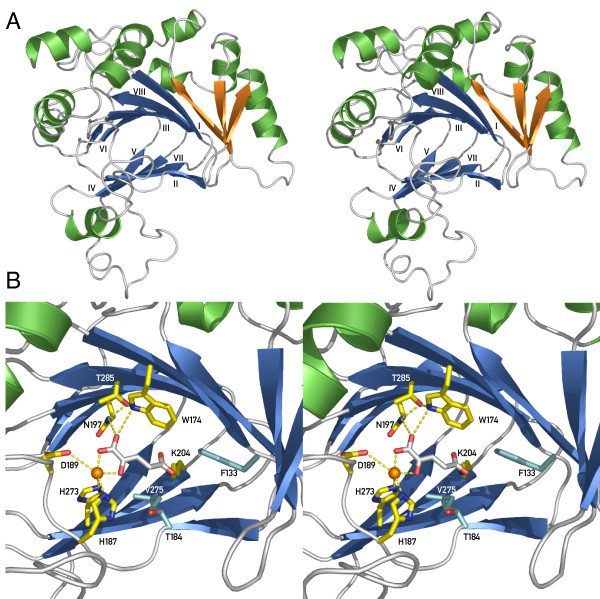
**Predicted structure of Jmjd6 based on comparative modelling (stereoview)**. Jmjd6 sequence alignments (Fig. 5) and HHpred were used to model Jmjd6 utilising the PDB structures of Hif1an (1h2k), Putative asparaginyl hydroxylase – 2636534 (1vrb) and Jmjd2a (2gp5), respectively. ***(A) ***Stereoview of the predicted structure of Jmjd6 presented as a ribbon model. The conserved eight-stranded DSBH core found in all Fe(II) and 2-oxoglutarate (2OG)-dependent dioxygenases is coloured in blue. Additional β-strands attached to the major β-sheet are shown in orange. Helices are depicted in green. ***(B) ***View of the predicted active site of the Jmjd6-Fe(II)-2OG complex showing coordination of Fe(II) to 2OG, His187, Asp189, and His273. 2OG also ligates to Trp174, Asn197 and Lys204 with Thr285 stabilising Asn197 and Trp174. Additional important residues for putative interactions are shown in cyan and include hydrophobic interactions from Phe133 and Val275 as well as Thr184, which is involved in 2OG-binding in Hif1an. Interacting residues along with the 2OG co-substrate are shown as stick presentations, putative H-bond interactions as dotted lines. Fe(II) is depicted as an orange ball.

Due to the fact that up to now no crystal structure of Jmjd6 is available, we decided to perform a homology/comparative modelling of the three-dimensional structure of the protein. The multiple sequence alignment of Jmjd6 homologous proteins (amino acids 44–329; Figure 5) was used for pairwise comparison of profile hidden Markov models (HMMs). This generated an alignment of the protein sequence that can be modelled with known related structures. This first step showed a significant probability (= 95%) of structural similarity of Jmjd6 to the three structurally resolved 2OG- and Fe(II)-dependent dioxygenases Hif1an (PDB entry 1h2k), putative asparaginyl hydroxylase – 2636534 from *Bacillus subtilis *(PDB entry 1vrb) and Jmjd2a (PDB entry 2gp5), respectively. The alignment generated by HHpred [74] was finally prepared to create a structural model and MODELLER [75] calculated this comparative model of the Jmjd6 protein based on the identified similarities containing all non-hydrogen atoms (Figure 6). The comparative modelling of Jmjd6 predicted that the Jmjd6 fold is a mixed alpha-beta structure composed of a major and a minor β-sheet surrounded by several α-helices. The structure showed the presence of the 2OG- and Fe(II)-dependent dioxygenase typical double-stranded β-helix (DSBH) motif as the protein core. It consists of two anti-parallel β-sheets composed of eight β-strands. The major sheet is defined by β-strands I, VIII, III, the minor sheet by β-strands VI and II, VII, IV, and V (Figure 6 and [see Additional file 7]), which form a jelly-roll topology. In addition, we recognised in the model three β-strands extending the core DSBH, resulting in a seven-stranded major β-sheet (green arrows in Figure 5 and orange arrows in Figure 6A). Interestingly, these additional strands were found to be paired anti-parallel to each other and to the major β-sheet.

Following iterative backbone annealing based on the structure of Hif1an (PDB entry 1h2k), 2OG and Fe(II) were added to the 3D model of Jmjd6. After energy minimisation utilising the SwissPDB Viewer, the predicted active site of the Jmjd6-Fe(II)-2OG complex was analysed in detail. Here, we found the proper positioning of the 2-His-1-carboxylate facial triad chelating the iron atom in a manner similar to other 2OG- and Fe(II)-dependent dioxygenases (Figure 6B). The HxD motif (His 187 and Asp 189) is predicted to be located just after the sequence of DSBH β-strand II and the second Fe(II)-binding histidine (His 273) is located on DSBH β-strand VII. The observation of Fe(II) coordination by these three residues confirms their initially suggested assignment based on the primary sequence analysis of the multiple sequence alignment of Jmjd6 proteins (Figure 5). In addition, we could identify in the model several residues within the putative active core of Jmjd6 capable of positioning the co-substrate 2OG in the cavity formed by the interior of the DSBH. The proper positioning of 2OG relative to the Fe(II) seems to be achieved by an interaction of several residues of the active core that are close enough to the hydrogen-bond with the 2OG. Among these residues we predict Asn197 and Trp174 to interact with the 1-carboxylate oxygen of 2OG that is not ligated to the Fe(II). Asn197 and Trp174 are stabilised by Thr285. Interestingly, in Jmjd6, like in Hif1an and Jmjd2a, a lysine (Lys204, from DSBH β-strand IV) could be important for the binding of the C5'-carboxylate of 2OG. Most of the other structurally resolved 2OG- and Fe(II)-dependent dioxygenases have instead of the threonine 285 an arginine at this position, which is part of a RXS motif within the DSBH β-strand VIII [76,77]. Thus, Jmjd6 has in contrast to other 2OG-dependent dioxygenases no tripeptide RXS motif (Figure 5). In addition to these major 2OG interacting residues in Jmjd6, we could recognise additional residues (Phe133 and Val275) for putative hydrophobic interactions in the active core of the protein.

**Figure 7:**
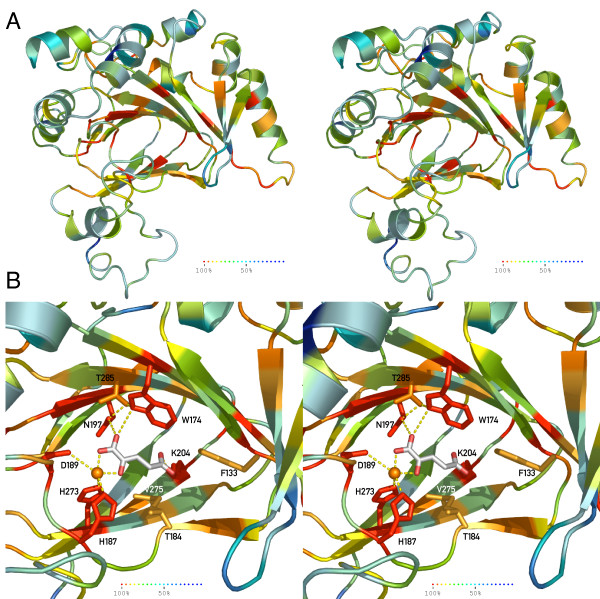
**Evolutional conservation of critical residues in Jmjd6 (stereoview)**. ***(A) ***Ribbon presentation of the predicted Jmjd6 structure is coloured based on the sequence homology observed in 54 Jmjd6 homologous proteins (Fig. 5). ***(B) ***Annotation of the sequence identity onto the catalytic domain demonstrated the conservation of the critical residues within the active site of the Jmjd6-Fe(II)-2OG complex. Residues were shaded according to the heat map with dark red representing total conservation of residues (100% sequence identity across 54 analysed species), and blue colour representing 50% sequence identity.

Beside the high probability of the Jmjd6 structure deduced from the comparative modelling approach, we searched for an additional, independent validation of this prediction. To achieve this, we decided to combine phylogenetic sequence analysis data and our comparative modelling data to proof the reliability of the Jmjd6 protein structure prediction. Accordingly, the amino acid identity across all 54 analysed species was translated into an indicative colour code ranging from dark red, representing amino acid identity across all species, to dark blue for highly variable/non-conserved residues (Figure 5). This colour code was applied to the calculated Jmjd6 structure and the individual amino acids were coloured according to their conservation (Figure 7). This computational approach demonstrated a strong conservation of the predicted Jmjd6 catalytic triad during evolution with 100% sequence identity of the HxD motif in all analysed 54 species (Figure 7A). In addition, residues that are predicted to bind 2-OG (Asn197, Thr285, Trp174, Lys204, and Thr187) are also highly conserved (Figure 7B), strongly suggesting that Jmjd6 is indeed possessing a 2OG-dependent dioxygenase function. Furthermore, a surface model of the protein with annotated sequence conservation shows that the putative active centre is located in a deep and narrow pocket [see Additional file 5]. Interestingly, the surface protein model also shows conservation of an exposed N-terminal region between β-strands 2 and 3 of the major β-sheet that is extending the DSBH core indicating that these residues might be important for interaction with other proteins [see Additional file 5]. In contrast to the sequence conservation observed in the predicted catalytic core and the exposed N-terminal major β-sheet, other regions of the protein have been much less conserved during evolution. Thus, the major selective pressure seems to have been on the catalytic function of the protein, while other areas of the protein retained flexibility in their functional design.

### Integration of Jmjd6 into the JmjC domain containing protein family

**Figure 8:**
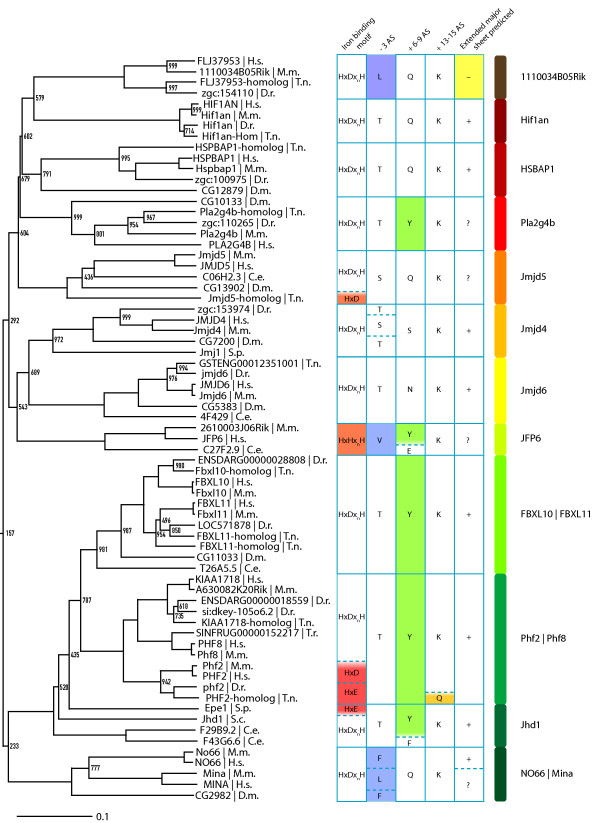
**Phylogenetic relationship of Jmjd6 to other JmjC domain containing proteins**. Detailed section of the phylogenetic tree of 313 JmjC proteins that is presented [in additional file 6]. Shown are 12 out of 25 identified DSBH fold containing protein subgroups as indicated with protein family names on the right side. Scale bar represents the relative phylogenetic distance as determined with PHYLIP. Bootstrap values are shown for values <1000. The first column of the table shows the residues potentially involved in iron binding. The second, third and fourth columns show potentially important residues within the -3, +6–8 and +13–15 regions in respect to the Hx(D/E/H) motif, respectively. Residues discussed in the text are highlighted in red, orange, blue, green and yellow. The fifth column shows the predicted ability to build an extended major sheet with three antiparallel β-strands as determined by Jpred. A "+" indicates the presence of three beta strands, a "?" two beta-strands and a "-" no predicted beta-strands within a 50 aa window starting 80 aa before the first β-strand from the JmjC jelly-roll. H.s. = *Homo sapiens*, M.m. = *Mus musculus*, D.r. = *Danio rerio*, T.n. = *Tetraodon nigroviridis*, T.r. = *Takifugu rubripes*, D.m. = *Drosophila melanogaster*, C.e. = *Caenorhabditis elegans*, S.c. = *Saccharomyces cerevisiae*, S.p. = *Schizosaccharomyces pombe*.

By using the computational pipeline described in Figure 1, we extracted a non-redundant set of 313 JmjC/DSBH domain-containing proteins from the Ensembl, Interpro, Uniprot and SMART databases. We selected these proteins from human and eight model organisms (mouse, zebrafish, pufferfish, tetraodon, nematode, fruitfly and yeast) based on the sequence homology of their JmjC domain to the mouse Jmjd6 and on the quality of the available sequence. Because genome-sequencing projects of many species are still ongoing, we had to exclude JmjC domain encoding genes from some species with low-sequence coverage genomes that did not match our quality criteria. Nevertheless, our comprehensive catalogue included 61 JmjC/DSBH-domain containing proteins from *Homo sapiens*, 57 from *Mus musculus*, 49 from *Danio rerio*, 53 from *Tetraodon *(*T. nigroviridis *and *T. rubripes*), 45 from *Drosophila melanogaster*, 27 from *Caenorhabditis elegans*, 6 from *Saccharomyces cerevisae*, and 10 from *Schizosaccharomyces pombe *[see Additional file 8]. The catalogue contains all classes of JmjC domain containing proteins that have been described so far, including "JmjC domain only" proteins. To classify these proteins into evolutionary subgroups and to investigate their evolutionary relationship we analysed the selected sequences. A multiple sequence alignment and subsequent bootstrapping analysis was carried out using ClustalW (1000 bootstrap trials, 111 seeds) and PHYLIP. Based on the similarity of the JmjC domain, the phylogenetic analysis revealed 25 evolutionary conserved subgroups [see Additional file 6]. These subgroups are in general accordance to previous studies, which reported the classification of 44 JmjC domain containing proteins into 4 related clusters [5] and of 98 JmjC domain containing proteins into 7 subfamilies [4]. Although, our analysis shows that especially the previously classified subgroup of "JmjC domain only family" [4] splits into 8 further subgroups based on the sequence divergence of their JmjC domain (1110034B05Rik family, Hif1an, Hspbap1, Pla2g4b, Jmjd5, Jmjd4, Jmjd6, JFP6-like, and No66/Mina53 subgroups). As previously described [4,5], these proteins have beside their JmjC domain no other recognisable protein domain but they are clearly functionally distinct from each other regarding reported protein interaction partners, cellular localisation and physiological functions. For example, Hif1an is an asparagine protein hydroxylase for the HIF1α transcription factor, Hspbap1 is a cytosolic protein that associates with heat shock protein 27 kD, and Jmjd6, Mina53, No66 are distinct nuclear proteins with as yet unknown molecular functions. In addition to these groups, we included in our analysis the AlkB, PH4/Phy-3, Ogfod2, Ogfod1/Tpa1, Plod 1–3, Lepre1/Leprel 1–2, 1110031I02Rik, and Egln1–3/Egl-9/Hph subgroups that were not analysed before together with the recently identified JmjC domain containing histone demethylase proteins [4]. Interestingly, we found that all subgroups could be classified independent of additional domain architectural features of the analysed proteins. As previously reported [4,5], the domain compositions of individual proteins classified into a given subgroup varied, indicating that neither their enzymatic specificity nor their functional relationships can be predicted solely based on the analysis of primary protein sequences. This is further supported by the identification of certain members within subgroups that obviously can not possess enzymatic functions as 2OG-dependent dioxygenases due to sequence changes within their predicted Fe(II) and/or 2OG-binding sites. For example, the Hairless protein within the Jmjd1/Hairless subgroup, the Epe1 protein in the Jhd1 subgroup and the whole Jarid2 protein subfamily contain substitutions in these binding sites indicating that these proteins are unlikely to be functional enzymes ([4] and Figure 8).

Interestingly, in some subgroups we observed a huge expansion in the number of family members. The P4H/Phy-3 family of prolyl-4-hydroxylases contained 22 proteins from *Drosophila melanogaster*. The P4H/Phy-3 subgroup compromises prolyl-4-hydroxylases involved in collagen biosynthesis or hypoxia sensing. Currently, it is unknown why especially the fly has so many members in this protein family. Another example is the AlkB subfamily of DNA and RNA demethylating enzymes. This group contains 45 proteins that split into the Alkbh1 to Alkbh8 protein family members. Apart from Alkbh1 to Alkbh3 that are known to be DNA/RNA repair enzymes [52,78,79] the exact biochemical activity of most of the other subfamily members has still to be determined.

Following phylogenetic analysis and tree generation a subtree encompassing the Jmjd6 protein was extracted for further analysis (Figure 8). By combining the information from the phylogenomic analysis with the annotation of protein subfamilies it is evident that Jmjd4 is closely related to Jmjd6 regarding sequence similarity. The JmjC domain of both proteins shows the highest sequence similarity among all 313 classified members (32% sequence identity and 50% sequence similarity). This indicates that *Jmjd4 *is the closest paralogue of *Jmjd6*.

Moreover, a detailed sequence analysis of JmjC domain containing protein subfamilies that are related to the Jmjd6 and Jmjd4 subgroups revealed further insights into Fe (II) and 2OG coordinating mechanisms (Figure 8). The JFP6 subgroup has a HxHxH motif instead of the Hx(D/E)x_n_H motif that is commonly used by all other JmjC domain containing proteins for the coordination of Fe(II) (Figure 8, orange box). If this motif is compatible with an oxygenase activity, as one would expect, has to be investigated in the future. Furthermore, the Jmjd5 homologue of *Tetraodon nigroviridis*, all Phf2 proteins, and Epe1 have only a Hx(D/E) motif (red box in Figure 8) and therefore lack the second histidine, which is known to be important for iron binding. Moreover, the *T*. *nigroviridis *Phf2 homologue lacks in addition the lysine that stabilises the C5'-caboxylate of 2OG. Next to this motif, Tuskada et al. described residues in positions -3, +6–8 and +13–15, in respect to the HxD motif to be important for the catalytic function of histone demethylases [21]. In the -3 position, the 11100034B05Rik, JFP6 and No66/Mina subgroups have a nonpolar side chain present in contrast to the polar side chains of serine/threonine residues in other subgroups. In the +6–8 position, members of the Pla2g4b, FBXL10/FBXL11, Phf2/Phf8, and individual members of the JFP6 and Jhd1 subgroup have a conserved tyrosine. In Epe1, this tyrosine has been shown to be important for heterochromatin destabilisation, which is independent of an Epe1 histone demethylation activity [21,46]. Another interesting feature is the predicted presence of an extended major β-sheet in most of the JmjC domain only protein families encompassing the subtree (Figure 8).

## Discussion

Here we report a systematic annotation and comparison of Jmjd6 homologous proteins across diverse phyla. With the recent description of the JmjC domain as a novel demethylase signature motif [21] and the discovery that many proteins containing JmjC domains are involved in reversing histone methylation marks [4,80], this protein superfamily has attracted central interest in many different fields of biology. It has become quite clear that these enzymes have not only important functions in the epigenetic regulation of chromatin modification states but also in diverse other physiological contexts ranging from oxygen sensing to fatty acid metabolism and DNA repair [81]. However, for most of these proteins it has remained difficult to unravel their molecular function or to predict their enzymatic targets solely based on sequence comparisons and analysis of their JmjC domains. In the past, we have been interested in the "phosphatidylserine receptor" (now renamed into jumonji domain containing 6), a JmjC domain containing protein, which was initially described to function as a type II transmembrane receptor for the engulfment of apoptotic cells [56]. To compare the JmjC domain of Jmjd6 to the DSBH/JmjC domains of other members within this protein superfamily we designed a semiautomated sequence retrieval and analysis routine that allowed us to establish a catalogue of 313 DSBH/JmjC domain containing proteins in nine selected species. In addition, we analysed the *Jmjd6 *and neighbouring loci in vertebrates and investigated their modes of expression.

### Comparative analysis of the vertebrate *Jmjd6 *locus and immediate neighbouring loci

With the ongoing genome sequencing projects in many different species and integrated efforts to annotate the functional content of fully sequenced genomes there is a need to complement existing automated genome annotations with a focused bioinformatics analysis of individual genes and gene families to make fully use of publicly available databases and resources. Such approaches might help to correct mistakes in automated annotation procedures and might add additional information to gene sets. This will finally lead to a more comprehensive and accurate annotation of gene functions.

We used the PipMaker alignment tool to analyse the patterns of sequence conservation across the mouse *Jmjd6 *and the neighbouring loci *1110005A03Rik *and *Sfrs2*. To identify all protein coding exons as well as highly conserved non-coding sequence elements in this region we analysed genomic sequences of 22 different vertebrate species. This led to the identification of an up to now overseen additional *Jmjd6 *exon in mammals and to the detection of a conserved putative bi-directional promoter element in between the closely spaced *Jmjd6 *and *1110005A03Rik *genes. The re-annotated genomic region encompassing the *Jmjd6*, *1110005A03Rik *and *Sfrs2 *loci was then the basis for a comparison of gene-targeting strategies that have been used to generate three different loss-of-function alleles of *Jmjd6 *in the mouse [54,62,63]. By aligning a map of restriction enzyme sites used for the construction of the different targeting vectors with the genomic region encompassing the three genes, we could show that in the *Jmjd6*^*tm*1*Flv *^allele and in the *Jmjd6*^*tm*1*Ysfk *^allele exon 1 of the *111005A03Rik *gene as well as the conserved intergenic region in between *Jmjd6 *and *111005A03Rik *was deleted upon homologous recombination in ES cells. An expression analysis of *Jmjd6*, *111005A03Rik *and *Sfsr2 *in the three different mouse alleles confirmed that *Jmjd6*^*tm*1*Flv *^and *Jmjd6*^*tm*1*Ysfk *^mutant mice are double knockouts of the *Jmjd6 *and *111005A03Rik *genes. In contrast, the *Jmjd6*^*tm*1*Gbf *^allele ablates only the expression of *Jmjd6*. Although all three independently generated knockout mice show to some degree overlapping phenotypes regarding defects during embryogenesis (e.g. disturbed eye development, brain malformations and defects in haematopoiesis) other controversial observations concerning the description of specific phenotypes have been reported [54,62,63]. Foremost, the capabilities of the mutant mouse lines to engulf apoptotic cells have been described differently. While Kunisaki et al. [63] and Li et al. [62] reported that their knockout mice showed impairment in the clearance of apoptotic cells during embryogenesis and defects in apoptotic cell phagocytosis assays, no such differences in phagocytosis competence could be detected in *Jmjd6*^*tm*1*Gbf *^mutant mice [54]. It has been suggested that differences in genetic backgrounds might account for these reported phenotypic variations [82-84]. However, in the light of the described results with *Jmjd6*^*tm*1*Flv *^and *Jmjd6*^*tm*1*Ysfk *^mutant mice being double knockouts of *Jmjd6 *and *111005A03Rik *the situation might be far more complex. It could well be that loss-of *111005A03Rik *function in *Jmjd6*^*tm*1*Flv *^and *Jmjd6*^*tm*1*Ysfk *^knockout mice might contribute in addition to the *Jmjd6*-deficiency to the phenotypic discrepancies observed. Future studies involving the generation and analysis of *111005A03Rik *knockout mice will address this possibility.

Interestingly, the as yet functionally uncharacterised *111005A03Rik *gene contains a predicted arginine protein methyltransferase domain. We demonstrate here, that the conserved sequence element in the short intergenic region between *Jmjd6 *and *111005A03Rik *possess bi-directional promoter activities for both genes. Of note, the forward orientation of the fragment, which controls *111005A03Rik *expression *in vivo *showed only half of the transcriptional activity as compared to the reverse orientation, which controls *Jmjd6 *expression *in vivo*. This indicates that both genes might compose a bi-directional transcriptional unit with opposing transcriptional and regulatory activities. This gives room for speculations. If *111005A03Rik *has a function as a methyltransferase and the Jmjd6 protein possess demethylation activity as many other JmjC domain containing proteins are doing, both proteins might be involved in a common biochemical pathway targeting possibly a shared substrate. Interestingly, such counterbalancing principles of methylation and demethylation activity have been already suggested for other JmjC domain containing proteins [53]. For example, the Alkbh8 protein contains an amino-terminal JmjC domain, which might possess DNA demethylase activity that is fused to a predicted C-terminal methylase domain. This domain architecture is conserved in the homologous Alkbh8 proteins in *Drosophila *(CG17807) and *C. elegans *(C14B1.10) suggesting the selection of a controlled methylation/demethylation regulatory mechanism during the evolution of these proteins.

If *Jmjd6 *and *111005A03Rik *also establish such counteracting functions needs to be addressed in the future. To proof such a hypothesis, the molecular function of both genes need first to be characterised. Nevertheless, the additional deletion of the *111005A03Rik *gene in two of the three described *Jmjd6 *knockout mice might well explain differences reported for their apoptotic cell engulfment phenotypes. If *111005A03Rik *is functioning as a methyltransferase in the control of transcriptional regulation, it is possible that the expression of other genes targeted or regulated by 111005A03Rik and/or Jmjd6 might influence phagocytosis of apoptotic cells. In such a scenario the described engulfment defects might be related to secondary effects caused by deregulation of genes involved in processes of phagocytosis or cellular differentiation of phagocytes.

Using PipMaker analysis based on sequence conservation we identified a before overseen exon in *Jmjd6 *loci of mammalian species. A subsequently undertaken e2g annotation of all available EST sequences to the *Jmjd6/JMJD6 *genomic regions lead to the discovery of two new alternative transcripts in mice and humans. At the protein level the most distal C-terminal part of the Jmjd6 protein (last 100 amino acids) is affected in all new identified transcripts. Either truncated polypeptides are generated that miss the poly-serine stretch, the putative AT-hook domain, and the predicted sumoylation and nuclear export signal (NES) or a stretch of eight amino acid is exchanged with 31 other residues. In this second splice variant identified in mice (Mm3) the predicted sumoylation site, the NES and the poly-serine stretch are retained but the putative AT-hook domain is missing (Figure 3C). Our 3D structural model of the Jmjd6 protein indicates that the sumoylation site, the NES and the AT-hook motif might be accessible for interacting proteins. The AT-hook domain is known to mediate binding to DNA in high-mobility group proteins [85]. Jmjd6 is a nuclear protein, which has been shown to contain five independently active nuclear localisation signals [58]. The NES might allow a shuttling of the protein from the nucleus to the cytoplasm. Likewise, the sumoylation site might be used for the regulation of interactions with other proteins.

A comparison of the Jmjd6 sequence with the resolved protein structure of the Hif1an/HIF1α C-terminal transactivation domain co-complex indicates that interactions with other proteins might occur in the Jmjd6 C-terminal region [49]. Alternatively, at this site homo-di- or homo-multimerization of Jmjd6 polypeptides might take place. For many JmjC/DSBH domain containing enzymes it is known that they can exist in various oligomeric forms. For example, Hif1an is a dimer in solution, germin forms a hexamer and HPPE a tetramer (for review see [6]). Indeed, very recently it could be demonstrated that Jmjd6 forms covalent multimers through homomeric polymerisation of the 50 kD protein [61]. Nuclear aggregated Jmjd6 products with masses of 100, 150, 200 and 250 kD were detected after immunoprecipitation indicating that assembly of homomultimers might be important for the protein function [61]. Thus, the new alternative transcripts encoded by the *Jmjd6 *locus might possess a regulatory role by suppressing multimer formation or interactions with other proteins. Such possibilities need to be experimentally investigated in the future.

### Structural model of the Jmjd6 protein and evolutionary relationship to other JmjC/DSBH-domain containing proteins

Recent crystallographic studies have helped to provide important insights in the structure and functions of JmjC domain containing proteins. More than ten structures of different members of this large oxygenase family have been solved demonstrating that the JmjC domain forms a characteristic DSBH structural fold composed of eight β-strands that build two β-sheets each containing four anti-parallel β-strands [6]. The catalytic core of these metalloenzymes consists of a highly conserved Hx(E/D)x_n_H signature motif that supplies three chelating positions for the Fe(II) iron and two additional chelating positions are contributed by the substrate co-factor 2OG. Very often the binding sites for 2OG in the DSBH core are as well conserved among the members of this protein family. Due to high sequence similarity of Jmjd6 to other JmjC domain containing proteins [57], we have embarked on the modelling of the Jmjd6 3D-structure to gain further insights into possible protein functions. Our analysis shows that Jmjd6 is indeed most likely a DSBH-fold containing 2OG-dependent dioxygenase enzyme, as previously first suggested by Cikala and colleagues [57]. All residues needed for Fe(II) coordination, 2OG co-substrate binding and oxidative catalysis were found to be located within the predicted DSBH domain of the protein model and are evolutionary preserved in all analysed Jmjd6 homologous proteins probably including those of non-metazoan species. We identified in all orthologous Jmjd6 proteins from metazoan species the characteristic HxDx_[n]_H catalytical triad and our model predicts five conserved residues (Trp174, Thr184, Asn197, Lys204, and Thr285) as being important for interaction with a 2OG co-substrate. In addition, the extreme conservation of residues forming the eight β-strands of the DSBH domain suggests a spatial and functional conservation of the Jmjd6 JmjC domain during evolution. This makes it highly likely that all Jmjd6 proteins in the different animal kingdoms are indeed active 2OG-dependent dioxygenase enzymes. This might not be true for other members of the JmjC/DSBH fold superfamily of proteins. For example, the subfamily of JFP6-like proteins has a substitution in the Fe(II) coordinating residues changing the Fe(II) binding motif. Furthermore, hairless and Jarid2 proteins are most likely enzymatically inactive due to the missing HxD motifs in their DSBH protein cores [4]. This suggests that JmjC domains might possess additional functions that are independent of any enzymatic activity.

2OG-dependent dioxygenases are known to catalyse two types of posttranslational protein modifications. Described have been hydroxylation of the amino acid side chains of proline, asparagines, aspartic acid and tryptophan, and secondly demethylation reactions on lysine residues (for review see [6,81]). It has been proposed that also methylated arginines might be targets of JmjC domain containing proteins. In fact, most recently it has been suggested that Jmjd6 might possess an enzymatic activity as an arginine histone demethylase. It was reported that Jmjd6 is capable to catalyse the removal of di-methyl groups on histone H3 at arginine 2 (H3R2) and on histone 4 at arginine 3 (H4R3) [86]. Asymmetric di-methylation of H3R2 has been associated with repression of transcriptional activity through its enrichment in heterochromatic loci and inactive euchromatic genes [87,88]. Thus, Jmjd6 might have a function in gene activation by erasing repressive chromatin marks. But it is also not unlikely that additional, non-histone substrates are targeted by Jmjd6.

All types of posttranslational modifications catalysed by 2OG-dependent dioxygenases involve a common type of hydroxylation reaction. During the catalytic reaction, the splitting of molecular oxygen is coupled to the hydroxylation of the target substrate and the oxidative decarboxylation of 2OG to give succinate and CO_2_. In the case of demethylation, hydroxylation of a methyl group is followed immediately by deformylation producing formaldehyde as an additional reaction product. As noted before by others, it is impossible to predict the prime substrate of 2OG-dependent dioxygenase based on a primary sequence analysis of the JmjC domain [80,81]. Even JmjC domain containing enzymes of the same subfamily such as the JHDM1A and the JHDM2A histone demethylases that both recognise H3K36 methylation have very limited primary or secondary structure similarity [22]. This suggests a requirement for an extended substrate recognition mechanism that probably involves protein-protein interactions in larger protein complexes or for a necessity of very defined interaction sites in the catalytic core that are important for determining substrate selectivity [89]. This is further supported by a number of recent studies that implicated other methyl-dependent protein domains like Tudor, MBT, PHD fingers and chromo domains as reader domains in the recognition of the histone code [90-94]. Jmjd6 contains a single JmjC domain and beside the predicted recognition sequences for nuclear in- and export, sumoylation and the AT-hook DNA-binding motif no other recognisable domain is present in the protein. Thus, the substrate specificity of Jmjd6 must be regulated by interaction with other proteins.

What is quite clear is that the putative enzymatic function of Jmjd6 as a 2OG-dependent dioxygenase is linked to its important role in development and morphogenesis, at least in vertebrates. All mouse mutants conclusively point to an essential role of *Jmjd6 *during embryogenesis were the gene is involved in the differentiation of multiple tissues and organs. Also in zebrafish, the morpholino knockdown of the *jmjd6 *orthologue resulted in severe defects in somite, brain, heart and notochord development [95]. This may or may not involve secondary effects on apoptosis induction and cell corpse clearance. Unlikely is a direct involvement of *jmjd6 *in apoptotic cell recognition or engulfment. Given that many 2OG-dependent dioxygenases have been implicated in the regulation of multiple pathways, global effects on gene expression and metabolism are not surprising when their enzymatic functions get perturbed or ablated.

The function of Jmjd6 during development seems to be different in vertebrates and invertebrates. Inactivation of the orthologous genes in *Drosophila *and *C. elegans *were surprisingly not associated with embryonic lethality and no major developmental defects have been reported for the loss-of-function mutants [59,96]. Interestingly, mutant flies that lack *dPSR *show inappropriate apoptosis during eye development [59]. Major phenotypes were observed in gain-of-function experiments in which the *dPSR *gene was overexpressed during development. Male transgenic flies that expressed *dPSR *under ubiquitous promoters developed a genital rotation phenotype. *dPSR *overexpression in the wing resulted in various wing blade fusion phenotypes and driving gene expression in the eye induced a small rough eye phenotype. Further genetic analysis pointed to an involvement of *dPSR *in the *head involution defective *(*hid*) and c-Jun-NH2 terminal kinase pathways. These suggested that loss of *dPSR *enhances apoptosis in the developing eye, while overexpression of *dPSR *protects from apoptosis. Of note, the analysis of mutant flies did not reveal a role for *dPSR *in the phagocytosis of apoptotic cells during development. Altogether, these observations support a function of *dPSR *in cell survival and differentiation that might have parallels in the vertebrate system [59].

When we compared the protein sequences of vertebrate and invertebrate orthologous Jmjd6 proteins we found divergence in the N- and C-terminal regions of the protein. Interestingly, the C-terminal poly-serine stretch is either shorter or missing in the invertebrate species. Moreover, we could not identify an additional exon in the invertebrate orthologous *Jmjd6 *genes that would generate alternative transcripts with effects on the C-terminal part of the protein. Therefore, it could well be that differences in this region of the protein are linked in vertebrates with different physiological functions of *Jmjd6 *protein, possibly through interactions with other proteins. This is also supported by identification of proteins in plants, fungi and protista that show partial sequence homology to the animalia Jmjd6 protein (Figure 5 and [see Additional file 4]). These were identified through sequence search routines focussing on a conserved N-terminal sequence stretch outside of the highly conserved JmjC domain. Consequently, these proteins show in their N-terminal parts significant sequence homology to the mouse Jmjd6 protein, but are diverse to Jmjd6 in their C-terminal regions. Moreover, many of these proteins are longer then the animalia Jmjd6 proteins (up to 2.000 aa instead of around 400 aa for animalia Jmjd6 proteins) and contain often in addition to the JmjC domain a single F-box domain.

Thus, it is very likely that they possess different or additional functions than the animalia Jmjd6 proteins and a possible homology of these proteins to Jmjd6 should be treated with caution. As the reciprocal high similarity suggests a relation to Jmjd6, it might be possible to postulate an ancestral "Jmjd6" gene that encoded a larger protein with additional domains (like F-box domains), which then have been lost during evolution of *Jmd6 *genes in animalia. Alternatively, the evolution of *Jmjd6 *genes in animalia could have been an independent event and these genes are not related in any way to the proteins we identified in plants, fungi and protista. In the future it might be possible to consolidate on one hypothesis, given that more functional data about these hypothetical proteins are being gathered and higher quality sequence information becomes available for phylogenetic analysis.

## Conclusion

Taken together, our integrated bioinformatic analysis provided new insights into possible structure-function relationships of Jmjd6 proteins and on the evolution of the whole superfamily of DSBH/JmjC domain containing proteins. Of note, the observations made on the protein model are not experimentally proven at the current state and await confirmation when the crystallographic structure of Jmjd6 has been obtained. In addition, we provide first evidence for a counteracting transcriptional regulative mechanism involving the co-localised *Jmjd6 *and *1110005A03Rik *genes.

## Methods

### Bioinformatic approaches

To investigate the splice variants of *Jmjd6*, the genomic sequence of *Jmjd6 *and adjacent genes on either side was extracted to perform an unrestricted e2g-based annotation of mouse ESTs with a seed-length of 18 and a minimum identity of 95% [67]. The resulting list of ESTs that overlap with the known *Jmjd6 *coding-region was passed on to a self-developed script that automatically retrieves the sequences of all matches and performs an in-depth verification of each EST using Sim4 [68]. The script sorts matches in different groups, according to the match quality. All matches with unidirectional splice sites, without internal gaps in the mRNA, and a <95% identity score to the corresponding genomic regions leaving aside the poly-A tail were used for manual annotation.

PipMaker sequence analysis [66] was done by extracting genomic sequences of 50 to 100 kb encompassing the *Jmjd6 *locus and neighbouring loci of mouse, human, bovine, armadillo, opossum, zebrafish, chicken, western clawed frog and puffer fish. Sequences were analysed by RepeatMasker [97] and positions of all coding sequences/exons in *Jmjd6*, *1110005A03Rik*, and *Sfrs2 *were identified and annotated using the BLAST algorithm. The masked and annotated mouse sequence were then finally used for interspecies pairwise alignment with the sequences from the different species mentioned above using the program PipMaker [66].

To identify *Jmjd6 *orthologous sequences, a standard NCBI blastp-search against the nr database using amino acids 52–152 of the murine *Jmjd6 *protein was performed [98]. Identified sequences were validated using the "reciprocal best BLAST hit" criterion in a *Mus musculus*-restricted blastp-search against the nr database [72].

SMART-parser, a Perl-script for batch queries against SMART [99], data retrieval and processing was developed to identify proteins with similar domains as Jmjd6. SMART and SMART-parser were used to automatically identify, extract and annotate the sequences of all JmjC-, Pfam-JmjC, Blast-JmjC-, P4Hc-, Pfam-2OG-FeII-Oxy- and Pfam-PhyH-domains for later processing. These domain names have been used in the different public databases to indicate the presence of the JmjC domain containing DSBH fold proteins.

Multiple sequence alignments were generated employing ClustalW with a default setup and five iterations of re-alignment. Visualisation, calculation of a background frequency corrected sequence logo, and calculation of amino acid conservation was done using LaTeX and TEXshade [100]. A ClustalW alignment of all Jmjd6 orthologues (amino acids 44–329) identified in the prior approaches was used to generate a 3D-structure of Jmjd6 using the pipeline from the MPI Toolkit [101]. To add 2OG and Fe(II) to the predicted structure and the predicted active site of Jmjd6, an iterative backbone annealing of the Hif1an structure (1h2k) on top of the Jmjd6 model was performed using the SwissPDB Viewer [102]. Afterwards, the coordinates of 2OG and Fe(II) were added to the Jmjd6 model and energies were minimised using DeepView. 3D representations of molecules were rendered using PyMOL [103].

For a maximum likelihood phylogenetic analysis of Jmjd6 orthologues, we employed the PHYLIP toolbox [104]. A ClustalW alignment of all proteins with significant homology to Jmjd6 was used as an input for ProML, using the Jones-Taylor-Thornton algorithm with an estimated constant rate of mutations, global rearrangements and the more accurate iterated branch lengths option. The primary protein sequences of Jmjd6/JMJD6 were analysed for sumoylation sites using a SUMOplot prediction with a cutt-off of 90% probability [105]. Nuclear export signals (NES) were identified using the NetNES 1.1 server using a cut-off of 95% identity to the NES Hidden Markow Model in the database [106]. Secondary structure predictions were generated using the Jpred web service [107].

### Identification of *Jmjd6 *splice variants

RNA was isolated from different cells (HEK 293-T, A549, mouse embryonic fibroblasts (MEFs) and reverse transcribed. Murine splice variants were amplified using the forward primer 5'-GGTGGTGCCTCTTCCCAACAAA-3' and the reverse primers 5'-TCCAGGTCAGGGTTGGGACAC-3' and 5'-AGCTAGAAGAGTCGCTGGAGCTGTC-3'. The amplicons of the primer pairs had a product size of 363 bp (Mm3), 407 bp (Mm2), and 432 bp (Mm1), respectively. All fragments were analysed by sequencing and the sequences were submitted to GenBank (Acc. No. EF527404 and EF527405). Human cDNAs (C1244142, C1244152, C1244171-10, C1244035-10, C1244149, C1244264-10, C1244183, C1244246-10, C1244122-10, C1244260-10) were obtained from the BioChain Institute (Hayward, USA) and amplified according to the manufactures instructions using the forward primer 5'-GGGAACTCATCAAAGTGACCCGAGACG-3' and the reverse primers 5'-ACTGCCGCTGCCCGTGCTGTATC-3' and 5'-CGAGTCTGCGAGGACTGCCAACTCG-3'. The amplicons had a product size of 320 bp (Hs2), 344 bp (Hs1) and 438 bp (Hs3), respectively. All fragments were analysed by sequencing and the sequences were submitted to GenBank (Acc. No. EF527406 and EF527407).

### Expression analysis of *Jmjd6*, *1110005A03Rik *and *Sfrs2*

RNA was isolated and reverse transcribed. Expression of *Jmjd6, 1110005A03Rik, Sfrs2 *in embryos or cells were analysed using the primers 5'-GTT CCA GCT CGT CAG ACT CG-3' and 5'-TGC CCC TAA GAC ATG ACC AC-3' for *Jmjd6*, 5'-GCC ACC ACA AGA CAT CAT TCT TG-3' and 5'-TCA GAT AAT TCA GCT TTA TGC CAG G-3' for *1110005A03Rik*, 5'-GCT CCA GAT CAA CCT CCA AG-3' and 5'-GCC ACC TGA GGC AGA TTA AA-3' for *Sfrs2*, respectively.

### Analysis of the bidirectional transcriptional unit

An 841 bp and an 840 bp fragment was amplified from mouse C57BL/6J genomic DNA using the forward 5'-CTCGAGCTCTCGTAGTAGTTGTGCCGGGT-3' and reverse 5'-GCTAGCACTGTCCCTAAATGTGTCACTGGAGC-3' primer as well as the forward 3'-GCTAGCTCTCGTAGTAGTTGTGCCGGGT-5' and reverse 5'-CTCGAGACTGTCCCTAAATGTGTCACTGGAGC-3' primer, respectively. These fragments were integrated into a promoter-less luciferase expression vector (pGL3, Promega, Madison, USA) and these constructs were introduced into HEK 293-T cells, A549 cells or mouse embryonic fibroblasts (MEFs) using Nanofection (PAA, Pasching, Austria). After 24h of cultivation at 37°C and 5% CO_2_, cell extracts were generated to measure the luciferase-activity using the Dual-Luciferase-Reporter-Assay System (Promega) according to the manufactures instructions. The obtained results were normalized to pGL4.74 (Promega) luciferase-activity. The measurements were performed in triplicate and repeated at least three times.

## Authors' contributions

PH and JB designed the computational pipeline flow together. The domain based multiple sequence alignment analysis of all protein sequences, the phylogeny prediction there from and the PipMaker sequence analysis as well as the analysis of the different mouse mutant *Jmjd6 *alleles was carried out by JB. PH contributed the full-length Jmjd6 analysis, 3D structure prediction and developed the new method for conservation based residue shading. Furthermore, PH identified and validated new splice variants of *Jmjd6*, characterised the bi-directional *Jmjd6 *transcriptional unit and developed SMART-parser as well as additional Perl scripts for automated data processing. SE carried out parts of the *Jmjd6 *expression analysis. AL coordinated the study and wrote the paper. All authors read and approved the final manuscript.

## Supplementary Material

Additional file 1 Validation of *JMJD6 *splices variants in humans. RT-PCR expression analysis of *JMJD6 *splice variants in a human cDNA tissue panel obtained from the BioChain Institute (Hayward, USA). Arrows indicate amplicons from correctly predicted splice variants that were confirmed by sequencing. Multiplex PCR amplification results in one verified unspecific PCR fragment migrating at approximately 400 bp. Amplification of the housekeeping gene *Ribosomal Protein 9 *(*RPS9*) was used as loading control. Click here for file

Additional file 2 Absence of nonsense-mediated decay in *Jmjd6 *splice variants. Two additional putative exons (green boxes) were identified **(A)**. Alternative splicing using these two exons results in the generation of two additional transcripts of Jmjd6 (variants 2 and 3, respectively). Half arrows and orange lines in the schematic presentation of the transcripts highlight the combination of exons detected by RT-PCR in **(B) **and **(C)**. Primers were designed to bind to the respective exons shown. **(B) **Experimental validation of the two new predicted alternative Jmjd6 transcripts using RT-PCR and agarose gel electrophoresis. Expression analyses confirmed that both splice variants are not subject to nonsense-mediated decay in adult mouse organs and **(C) **human tissues. Amplification of the housekeeping β-actin gene was used as a RNA loading control. Click here for file

Additional file 3 Comparison of strategies used to inactivate the *Jmjd6 *gene in mouse ES-cells. Schematic representation of the mouse locus according to the Ensembl annotation of the *Jmjd6 *gene and its neighbouring genes ***(A)***. Arrows indicate transcriptional orientation of the genes along chromosome 11, from centromer to telomer. *Jmjd6 *exons are shown in green, exons of *1110005A03Rik *in blue, and of *Sfrs2 *in yellow. Individual exons are numbered. The strategies used to generate the different targeted knock out alleles are shown. Red boxes indicate gene regions deleted by homologous recombination. The *Jmjd6*^*tm*1*Flv *^allele was generated by replacing an *Aat*II/*Spe*I fragment by a neomycin resistance cassette. In the *Jmjd6*^*tm*1*Ysfk *^allele the *Eco*RI fragment and in the *Jmjd6*^*tm*1*Gbf *^allele the *Aat*II/*Rsr*II fragment were replaced respectively. Based on these chosen fragments, targeted inactivation of *Jmjd6 *seems to affect in two of the three alleles the neighbouring *1110005A03Rik *locus. ***(B) ***RT-PCR analysis of the expression of the *Jmjd6*, *1110005A03Rik *(*Rik*) and *Sfrs2 *genes in *Jmjd6 *wild type and *Jmjd6 *homozygous mutant embryos. The expression of *1110005A03Rik *is not detectable in the *Jmjd6*^*tm*1*Flv *^allele and in the *Jmjd6*^*tm*1*Ysfk *^allele, whereas expression of *Sfrs2 *is unaltered in all three mouse lines investigated ***(C) ***Semi-quantitative RT-PCR analysis of *1110005A03Rik *expression in *Jmjd6*^*tm*1*Gbf *^wild type and homozygous mutant embryos (embryonic stage E13.5) and in fetal-liver derived macrophages shows that the level of expression is not altered due to the targeted inactivation of the *Jmjd6 *gene in this mouse line. RT-PCR expression analysis was performed in differential dilution steps of cDNA material (1, 1:10, 1:100). In all RT-PCR expression experiments amplification of the housekeeping gene *β-actin *severed as loading control. Click here for file

Additional file 4 Phylogenetic tree of live for identified putative Jmjd6 orthologs and their domain composition. Scientific classification of species with identified putative Jmjd6 orthologs was performed according to the NCBI taxonomy browser [108]. Schematic presentation of the grouping and categorisation of 62 species with identified Jmjd6 proteins. The individual species are shown on the left side, underlying colours highlight the kingdoms – animalia (red), plantae (green), protista (blue), fungi (yellow), and eubacteria (magenta), boxes on the right side represent the domain. The intermediate boxes show informative higher order ranks (e.g. genus, family, order, class, or phyla). On the left side, the domain composition for each protein is given. Length of polypeptides in amino acids (aa) are indicated on the top. Asterisks indicate partial or truncated protein sequences. Click here for file

Additional file 9 Jmjd6 homologous proteins. Catalogue of 62 identified Jmjd6 homologous proteins. Click here for file

Additional file 7 Features of structurally resolved 2OG-Fe(II)-dependent dioxygenases in comparison to Jmjd6. Table describing features of structurally resolved and experimentally characterised 2OG-Fe(II)-dependent dioxygenases in comparison to Jmjd6. Click here for file

Additional file 5 Evolutionary conservation of surface exposed residues in the Jmjd6 protein model (stereoview). The surface model was computed using PyMol and coloured according to the sequence conservation code below in percent sequence identity in 54 analysed species. The coordinating Fe(II) of the catalytic triad is depicted in black, the co-substrate 2OG is shown as stick representation. Click here for file

Additional file 6 Phylogenetic relationship of Jmjd6 to the superfamily of JmjC-domain containing proteins. In total 313 sequences of DSBH/JmjC domain containing proteins from humans and model organisms (mouse, zebrafish, pufferfish, nematode, fruit fly, and yeast) were analysed for their relationship to different subgroups of JmjC domain containing proteins. JmjC domain sequences of the proteins were analysed by multiple sequence alignment and subsequent bootstrap analysis using ClustalW (1000 bootstrap trials, 111 seeds) and PHYLIP (ProML). The resulting unrouted tree based on sequence similarity within the JmjC domain revealed 25 evolutionary conserved protein subgroups. These are highlighted in different colours according to the colour code on the left side. Five proteins that could not be grouped into subgroups are indicated in red letters. H.s. = *Homo sapiens*, M.m. = *Mus musculus*, D.r. = *Danio rerio*, T.n. = *Tetraodon nigroviridis*, T.r. = *Takifugu rubripes*, D.m. = *Drosophila melanogaster*, C.e. = *Caenorhabditis elegans*, S.c. = *Saccharomyces cerevisiae*, S.p. = *Schizosaccharomyces pombe*. Click here for file

Additional file 8 Catalogue of JmjC/DSBH domain containing proteins. Table of 313 JmjC/DSBH domain containing protein used for the phylogenetic analysis. Click here for file

## References

[B1] Takeuchi T, Yamazaki Y, Katoh-Fukui Y, Tsuchiya R, Kondo S, Motoyama J, Higashinakagawa T (1995). Gene trap capture of a novel mouse gene, jumonji, required for neural tube formation. Genes Dev.

[B2] Balciunas D, Ronne H (2000). Evidence of domain swapping within the jumonji family of transcription factors. Trends Biochem Sci.

[B3] Clissold PM, Ponting CP (2001). JmjC: cupin metalloenzyme-like domains in jumonji, hairless and phospholipase A2beta. Trends Biochem Sci.

[B4] Klose RJ, Kallin EM, Zhang Y (2006). JmjC-domain-containing proteins and histone demethylation. Nat Rev Genet.

[B5] Takeuchi T, Watanabe Y, Takano-Shimizu T, Kondo S (2006). Roles of jumonji and jumonji family genes in chromatin regulation and development. Dev Dyn.

[B6] Clifton IJ, McDonough MA, Ehrismann D, Kershaw NJ, Granatino N, Schofield CJ (2006). Structural studies on 2-oxoglutarate oxygenases and related double-stranded beta-helix fold proteins. J Inorg Biochem.

[B7] Dunwell JM, Culham A, Carter CE, Sosa-Aguirre CR, Goodenough PW (2001). Evolution of functional diversity in the cupin superfamily. Trends Biochem Sci.

[B8] Schofield CJ, Zhang Z (1999). Structural and mechanistic studies on 2-oxoglutarate-dependent oxygenases and related enzymes. Curr Opin Struct Biol.

[B9] Trewick SC, McLaughlin PJ, Allshire RC (2005). Methylation: lost in hydroxylation?. EMBO Rep.

[B10] Lee Y, Song AJ, Baker R, Micales B, Conway SJ, Lyons GE (2000). Jumonji, a nuclear protein that is necessary for normal heart development. Circ Res.

[B11] Takeuchi T, Kojima M, Nakajima K, Kondo S (1999). jumonji gene is essential for the neurulation and cardiac development of mouse embryos with a C3H/He background. Mech Dev.

[B12] Toyoda M, Kojima M, Takeuchi T (2000). Jumonji is a nuclear protein that participates in the negative regulation of cell growth. Biochem Biophys Res Commun.

[B13] Benevolenskaya EV, Murray HL, Branton P, Young RA, Kaelin WG (2005). Binding of pRB to the PHD protein RBP2 promotes cellular differentiation. Mol Cell.

[B14] Hsieh JC, Sisk JM, Jurutka PW, Haussler CA, Slater SA, Haussler MR, Thompson CC (2003). Physical and functional interaction between the vitamin D receptor and hairless corepressor, two proteins required for hair cycling. J Biol Chem.

[B15] Moraitis AN, Giguere V, Thompson CC (2002). Novel mechanism of nuclear receptor corepressor interaction dictated by activation function 2 helix determinants. Mol Cell Biol.

[B16] Potter GB, Beaudoin GM, DeRenzo CL, Zarach JM, Chen SH, Thompson CC (2001). The hairless gene mutated in congenital hair loss disorders encodes a novel nuclear receptor corepressor. Genes Dev.

[B17] Ahmad W, Faiyaz ul Haque M, Brancolini V, Tsou HC, ul Haque S, Lam H, Aita VM, Owen J, deBlaquiere M, Frank J (1998). Alopecia universalis associated with a mutation in the human hairless gene. Science.

[B18] Cichon S, Anker M, Vogt IR, Rohleder H, Putzstuck M, Hillmer A, Farooq SA, Al-Dhafri KS, Ahmad M, Haque S (1998). Cloning, genomic organization, alternative transcripts and mutational analysis of the gene responsible for autosomal recessive universal congenital alopecia. Hum Mol Genet.

[B19] Zarach JM, Beaudoin GM, Coulombe PA, Thompson CC (2004). The co-repressor hairless has a role in epithelial cell differentiation in the skin. Development.

[B20] Martin C, Zhang Y (2005). The diverse functions of histone lysine methylation. Nat Rev Mol Cell Biol.

[B21] Tsukada Y, Fang J, Erdjument-Bromage H, Warren ME, Borchers CH, Tempst P, Zhang Y (2006). Histone demethylation by a family of JmjC domain-containing proteins. Nature.

[B22] Chen Z, Zang J, Whetstine J, Hong X, Davrazou F, Kutateladze TG, Simpson M, Mao Q, Pan CH, Dai S (2006). Structural insights into histone demethylation by JMJD2 family members. Cell.

[B23] Whetstine JR, Nottke A, Lan F, Huarte M, Smolikov S, Chen Z, Spooner E, Li E, Zhang G, Colaiacovo M, Shi Y (2006). Reversal of histone lysine trimethylation by the JMJD2 family of histone demethylases. Cell.

[B24] Klose RJ, Gardner KE, Liang G, Erdjument-Bromage H, Tempst P, Zhang Y (2007). Demethylation of histone H3K36 and H3K9 by Rph1: a vestige of an H3K9 methylation system in Saccharomyces cerevisiae?. Mol Cell Biol.

[B25] Tu S, Bulloch EM, Yang L, Ren C, Huang WC, Hsu PH, Chen CH, Liao CL, Yu HM, Lo WS (2007). Identification of histone demethylases in Sacchromyces cerevisiae. J Biol Chem.

[B26] Yamane K, Tateishi K, Klose RJ, Fang J, Fabrizio LA, Erdjument-Bromage H, Taylor-Papadimitriou J, Tempst P, Zhang Y (2007). PLU-1 is an H3K4 demethylase involved in transcriptional repression and breast cancer cell proliferation. Mol Cell.

[B27] Cloos PA, Christensen J, Agger K, Maiolica A, Rappsilber J, Antal T, Hansen KH, Helin K (2006). The putative oncogene GASC1 demethylates tri- and dimethylated lysine 9 on histone H3. Nature.

[B28] Fodor BD, Kubicek S, Yonezawa M, O'Sullivan RJ, Sengupta R, Perez-Burgos L, Opravil S, Mechtler K, Schotta G, Jenuwein T (2006). Jmjd2b antagonizes H3K9 trimethylation at pericentric heterochromatin in mammalian cells. Genes Dev.

[B29] Klose RJ, Yamane K, Bae Y, Zhang D, Erdjument-Bromage H, Tempst P, Wong J, Zhang Y (2006). The transcriptional repressor JHDM3A demethylates trimethyl histone H3 lysine 9 and lysine 36. Nature.

[B30] Wissmann M, Yin N, Muller JM, Greschik H, Fodor BD, Jenuwein T, Vogler C, Schneider R, Gunther T, Buettner R (2007). Cooperative demethylation by JMJD2C and LSD1 promotes androgen receptor-dependent gene expression. Nat Cell Biol.

[B31] Christensen J, Agger K, Cloos PA, Pasini D, Rose S, Sennels L, Rappsilber J, Hansen KH, Salcini AE, Helin K (2007). RBP2 belongs to a family of demethylases, specific for tri-and dimethylated lysine 4 on histone 3. Cell.

[B32] Klose RJ, Yan Q, Tothova Z, Yamane K, Erdjument-Bromage H, Tempst P, Gilliland DG, Zhang Y, Kaelin WG (2007). The retinoblastoma binding protein RBP2 is an H3K4 demethylase. Cell.

[B33] Iwase S, Lan F, Bayliss P, de la Torre-Ubieta L, Huarte M, Qi HH, Whetstine JR, Bonni A, Roberts TM, Shi Y (2007). The X-linked mental retardation gene SMCX/JARID1C defines a family of histone H3 lysine 4 demethylases. Cell.

[B34] Lee MG, Norman J, Shilatifard A, Shiekhattar R (2007). Physical and functional association of a trimethyl H3K4 demethylase and Ring6a/MBLR, a polycomb-like protein. Cell.

[B35] Liang G, Klose RJ, Gardner KE, Zhang Y (2007). Yeast Jhd2p is a histone H3 Lys4 trimethyl demethylase. Nat Struct Mol Biol.

[B36] Seward DJ, Cubberley G, Kim S, Schonewald M, Zhang L, Tripet B, Bentley DL (2007). Demethylation of trimethylated histone H3 Lys4 in vivo by JARID1 JmjC proteins. Nat Struct Mol Biol.

[B37] Eissenberg JC, Lee MG, Schneider J, Ilvarsonn A, Shiekhattar R, Shilatifard A (2007). The trithorax-group gene in Drosophila little imaginal discs encodes a trimethylated histone H3 Lys4 demethylase. Nat Struct Mol Biol.

[B38] Lee N, Zhang J, Klose RJ, Erdjument-Bromage H, Tempst P, Jones RS, Zhang Y (2007). The trithorax-group protein Lid is a histone H3 trimethyl-Lys4 demethylase. Nat Struct Mol Biol.

[B39] Secombe J, Li L, Carlos L, Eisenman RN (2007). The Trithorax group protein Lid is a trimethyl histone H3K4 demethylase required for dMyc-induced cell growth. Genes Dev.

[B40] Huarte M, Lan F, Kim T, Vaughn MW, Zaratiegui M, Martienssen RA, Buratowski S, Shi Y (2007). The fission yeast JMJ2 reverses histone H3 lysine 4 tri-methylation. J Biol Chem.

[B41] Agger K, Cloos PA, Christensen J, Pasini D, Rose S, Rappsilber J, Issaeva I, Canaani E, Salcini AE, Helin K (2007). UTX and JMJD3 are histone H3K27 demethylases involved in HOX gene regulation and development. Nature.

[B42] De Santa F, Totaro MG, Prosperini E, Notarbartolo S, Testa G, Natoli G (2007). The histone H3 lysine-27 demethylase Jmjd3 links inflammation to inhibition of polycomb-mediated gene silencing. Cell.

[B43] Lan F, Bayliss PE, Rinn JL, Whetstine JR, Wang JK, Chen S, Iwase S, Alpatov R, Issaeva I, Canaani E (2007). A histone H3 lysine 27 demethylase regulates animal posterior development. Nature.

[B44] Lee MG, Villa R, Trojer P, Norman J, Yan KP, Reinberg D, Di Croce L, Shiekhattar R (2007). Demethylation of H3K27 regulates polycomb recruitment and H2A ubiquitination. Science.

[B45] Yamane K, Toumazou C, Tsukada Y, Erdjument-Bromage H, Tempst P, Wong J, Zhang Y (2006). JHDM2A, a JmjC-containing H3K9 demethylase, facilitates transcription activation by androgen receptor. Cell.

[B46] Zofall M, Grewal SI (2006). Swi6/HP1 recruits a JmjC domain protein to facilitate transcription of heterochromatic repeats. Mol Cell.

[B47] Huang Y, Fang J, Bedford MT, Zhang Y, Xu RM (2006). Recognition of histone H3 lysine-4 methylation by the double tudor domain of JMJD2A. Science.

[B48] Dann CE, Bruick RK, Deisenhofer J (2002). Structure of factor-inhibiting hypoxia-inducible factor 1: An asparaginyl hydroxylase involved in the hypoxic response pathway. Proc Natl Acad Sci USA.

[B49] Elkins JM, Hewitson KS, McNeill LA, Seibel JF, Schlemminger I, Pugh CW, Ratcliffe PJ, Schofield CJ (2003). Structure of factor-inhibiting hypoxia-inducible factor (HIF) reveals mechanism of oxidative modification of HIF-1 alpha. J Biol Chem.

[B50] Schofield CJ, Ratcliffe PJ (2004). Oxygen sensing by HIF hydroxylases. Nat Rev Mol Cell Biol.

[B51] Falnes PO, Johansen RF, Seeberg E (2002). AlkB-mediated oxidative demethylation reverses DNA damage in Escherichia coli. Nature.

[B52] Trewick SC, Henshaw TF, Hausinger RP, Lindahl T, Sedgwick B (2002). Oxidative demethylation by Escherichia coli AlkB directly reverts DNA base damage. Nature.

[B53] Aravind L, Koonin EV (2001). The DNA-repair protein AlkB, EGL-9, and leprecan define new families of 2-oxoglutarate- and iron-dependent dioxygenases. Genome Biol.

[B54] Böse J, Gruber AD, Helming L, Schiebe S, Wegener I, Hafner M, Beales M, Kontgen F, Lengeling A (2004). The phosphatidylserine receptor has essential functions during embryogenesis but not in apoptotic cell removal. J Biol.

[B55] Schneider JE, Böse J, Bamforth SD, Gruber AD, Broadbent C, Clarke K, Neubauer S, Lengeling A, Bhattacharya S (2004). Identification of cardiac malformations in mice lacking Ptdsr using a novel high-throughput magnetic resonance imaging technique. BMC Dev Biol.

[B56] Fadok VA, Bratton DL, Rose DM, Pearson A, Ezekewitz RA, Henson PM (2000). A receptor for phosphatidylserine-specific clearance of apoptotic cells. Nature.

[B57] Cikala M, Alexandrova O, David CN, Proschel M, Stiening B, Cramer P, Böttger A (2004). The phosphatidylserine receptor from Hydra is a nuclear protein with potential Fe(II) dependent oxygenase activity. BMC Cell Biol.

[B58] Cui P, Qin B, Liu N, Pan G, Pei D (2004). Nuclear localization of the phosphatidylserine receptor protein via multiple nuclear localization signals. Exp Cell Res.

[B59] Krieser RJ, Moore FE, Dresnek D, Pellock BJ, Patel R, Huang A, Brachmann C, White K (2007). The Drosophila homolog of the putative phosphatidylserine receptor functions to inhibit apoptosis. Development.

[B60] Mitchell JE, Cvetanovic M, Tibrewal N, Patel V, Colamonici OR, Li MO, Flavell RA, Levine JS, Birge RB, Ucker DS (2006). The presumptive phosphatidylserine receptor is dispensable for innate anti-inflammatory recognition and clearance of apoptotic cells. J Biol Chem.

[B61] Tibrewal N, Liu T, Li H, Birge RB (2007). Characterization of the biochemical and biophysical properties of the phosphatidylserine receptor (PS-R) gene product. Mol Cell Biochem.

[B62] Li MO, Sarkisian MR, Mehal WZ, Rakic P, Flavell RA (2003). Phosphatidylserine receptor is required for clearance of apoptotic cells. Science.

[B63] Kunisaki Y, Masuko S, Noda M, Inayoshi A, Sanui T, Harada M, Sasazuki T, Fukui Y (2004). Defective fetal liver erythropoiesis and T lymphopoiesis in mice lacking the phosphatidylserine receptor. Blood.

[B64] Fackelmayer FO (2005). Protein arginine methyltransferases: guardians of the Arg?. Trends Biochem Sci.

[B65] Bermingham JR, Arden KC, Naumova AK, Sapienza C, Viars CS, Fu XD, Khotz J, Manley JL, Rosenfeld MG (1995). Chromosomal localization of mouse and human genes encoding the splicing factors ASF/SF2 (SFRS1) and SC-35 (SFRS2). Genomics.

[B66] Schwartz S, Zhang Z, Frazer KA, Smit A, Riemer C, Bouck J, Gibbs R, Hardison R, Miller W (2000). PipMaker – a web server for aligning two genomic DNA sequences. Genome Res.

[B67] Krüger J, Sczyrba A, Kurtz S, Giegerich R (2004). e2g: an interactive web-based server for efficiently mapping large EST and cDNA sets to genomic sequences. Nucleic Acids Res.

[B68] Florea L, Hartzell G, Zhang Z, Rubin GM, Miller W (1998). A computer program for aligning a cDNA sequence with a genomic DNA sequence. Genome Res.

[B69] Fadok VA, Xue D, Henson P (2001). If phosphatidylserine is the death knell, a new phosphatidylserine-specific receptor is the bellringer. Cell Death Differ.

[B70] Huntley MA, Golding GB (2006). Selection and slippage creating serine homopolymers. Mol Biol Evol.

[B71] Amrani N, Sachs MS, Jacobson A (2006). Early nonsense: mRNA decay solves a translational problem. Nat Rev Mol Cell Biol.

[B72] Tatusov RL, Koonin EV, Lipman DJ (1997). A genomic perspective on protein families. Science.

[B73] Hubbard TJ, Aken BL, Beal K, Ballester B, Caccamo M, Chen Y, Clarke L, Coates G, Cunningham F, Cutts T (2007). Ensembl 2007. Nucleic Acids Res.

[B74] Söding J (2005). Protein homology detection by HMM-HMM comparison. Bioinformatics.

[B75] Sali A, Blundell TL (1993). Comparative protein modelling by satisfaction of spatial restraints. J Mol Biol.

[B76] Britsch L, Dedio J, Saedler H, Forkmann G (1993). Molecular characterization of flavanone 3 beta-hydroxylases. Consensus sequence, comparison with related enzymes and the role of conserved histidine residues. Eur J Biochem.

[B77] Hogan DA, Smith SR, Saari EA, McCracken J, Hausinger RP (2000). Site-directed mutagenesis of 2,4-dichlorophenoxyacetic acid/alpha-ketoglutarate dioxygenase. Identification of residues involved in metallocenter formation and substrate binding. J Biol Chem.

[B78] Lee DH, Jin SG, Cai S, Chen Y, Pfeifer GP, O'Connor TR (2005). Repair of methylation damage in DNA and RNA by mammalian AlkB homologues. J Biol Chem.

[B79] Yu B, Edstrom WC, Benach J, Hamuro Y, Weber PC, Gibney BR, Hunt JF (2006). Crystal structures of catalytic complexes of the oxidative DNA/RNA repair enzyme AlkB. Nature.

[B80] Klose RJ, Zhang Y (2007). Regulation of histone methylation by demethylimination and demethylation. Nat Rev Mol Cell Biol.

[B81] Ozer A, Bruick RK (2007). Non-heme dioxygenases: cellular sensors and regulators jelly rolled into one?. Nat Chem Biol.

[B82] Weitzman JB (2004). The curious world of apoptotic cell clearance. J Biol.

[B83] Williamson P, Schlegel RA (2004). Hide and seek: the secret identity of the phosphatidylserine receptor. J Biol.

[B84] Wolf A, Schmitz C, Bottger A (2007). Changing story of the receptor for phosphatidylserine-dependent clearance of apoptotic cells. EMBO Rep.

[B85] Aravind L, Landsman D (1998). AT-hook motifs identified in a wide variety of DNA-binding proteins. Nucleic Acids Res.

[B86] Chang B, Chen Y, Zhao Y, Bruick RK (2007). JMJD6 is a histone arginine demethylase. Science.

[B87] Guccione E, Bassi C, Casadio F, Martinato F, Cesaroni M, Schuchlautz H, Luscher B, Amati B (2007). Methylation of histone H3R2 by PRMT6 and H3K4 by an MLL complex are mutually exclusive. Nature.

[B88] Kirmizis A, Santos-Rosa H, Penkett CJ, Singer MA, Vermeulen M, Mann M, Bahler J, Green RD, Kouzarides T (2007). Arginine methylation at histone H3R2 controls deposition of H3K4 trimethylation. Nature.

[B89] Ng SS, Kavanagh KL, McDonough MA, Butler D, Pilka ES, Lienard BM, Bray JE, Savitsky P, Gileadi O, von Delft F (2007). Crystal structures of histone demethylase JMJD2A reveal basis for substrate specificity. Nature.

[B90] Kim J, Daniel J, Espejo A, Lake A, Krishna M, Xia L, Zhang Y, Bedford MT (2006). Tudor, MBT and chromo domains gauge the degree of lysine methylation. EMBO Rep.

[B91] Li H, Ilin S, Wang W, Duncan EM, Wysocka J, Allis CD, Patel DJ (2006). Molecular basis for site-specific read-out of histone H3K4me3 by the BPTF PHD finger of NURF. Nature.

[B92] Pena PV, Davrazou F, Shi X, Walter KL, Verkhusha VV, Gozani O, Zhao R, Kutateladze TG (2006). Molecular mechanism of histone H3K4me3 recognition by plant homeodomain of ING2. Nature.

[B93] Shi X, Hong T, Walter KL, Ewalt M, Michishita E, Hung T, Carney D, Pena P, Lan F, Kaadige MR (2006). ING2 PHD domain links histone H3 lysine 4 methylation to active gene repression. Nature.

[B94] Wysocka J, Swigut T, Xiao H, Milne TA, Kwon SY, Landry J, Kauer M, Tackett AJ, Chait BT, Badenhorst P (2006). A PHD finger of NURF couples histone H3 lysine 4 trimethylation with chromatin remodelling. Nature.

[B95] Hong JR, Lin GH, Lin CJ, Wang WP, Lee CC, Lin TL, Wu JL (2004). Phosphatidylserine receptor is required for the engulfment of dead apoptotic cells and for normal embryonic development in zebrafish. Development.

[B96] Wang X, Wu YC, Fadok VA, Lee MC, Gengyo-Ando K, Cheng LC, Ledwich D, Hsu PK, Chen JY, Chou BK (2003). Cell corpse engulfment mediated by C. elegans phosphatidylserine receptor through CED-5 and CED-12. Science.

[B97] RepeatMasker. http://www.repeatmasker.org.

[B98] NCBI-BLAST. http://www.ncbi.nlm.nih.gov/BLAST.

[B99] SMART. http://smart.embl-heidelberg.de.

[B100] Beitz E (2000). TEXshade: shading and labeling of multiple sequence alignments using LATEX2 epsilon. Bioinformatics.

[B101] Biegert A, Mayer C, Remmert M, Soding J, Lupas AN (2006). The MPI Bioinformatics Toolkit for protein sequence analysis. Nucleic Acids Res.

[B102] Swiss-Pdb-Viewer. http://expasy.org/spdbv.

[B103] PyMOL. http://pymol.sourceforge.net/.

[B104] PHYLIP. http://evolution.genetics.washington.edu/phylip.html.

[B105] SUMOplot. http://www.abgent.com.cn/doc/sumoplot/login.asp.

[B106] NetNES. http://www.cbs.dtu.dk/services/NetNES.

[B107] Jpred-3. http://www.compbio.dundee.ac.uk/~www-jpred.

[B108] NCBI-Taxonomy. http://www.ncbi.nlm.nih.gov/Taxonomy/.

