# Supplementary figures and images for: Genomic structure and expression of Jmjd6 and evolutionary analysis in the context of related JmjC domain containing proteins
Source: BMC Genomics. 2008 Jun 18;9:293. doi: 10.1186/1471-2164-9-293 (PMC2453528; doi:10.1186/1471-2164-9-293)

A

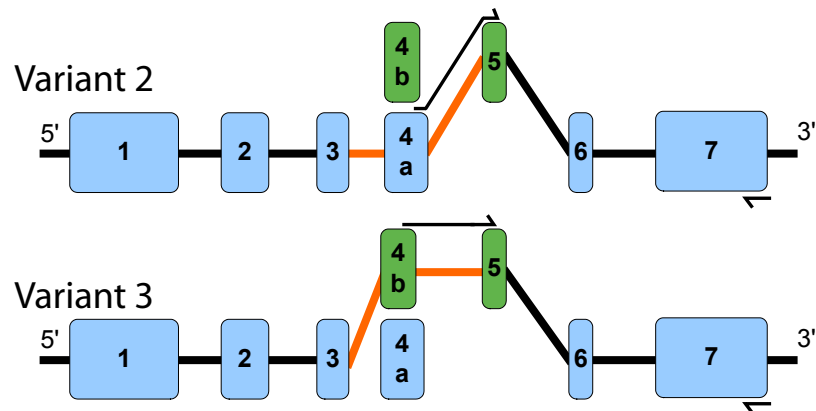

C

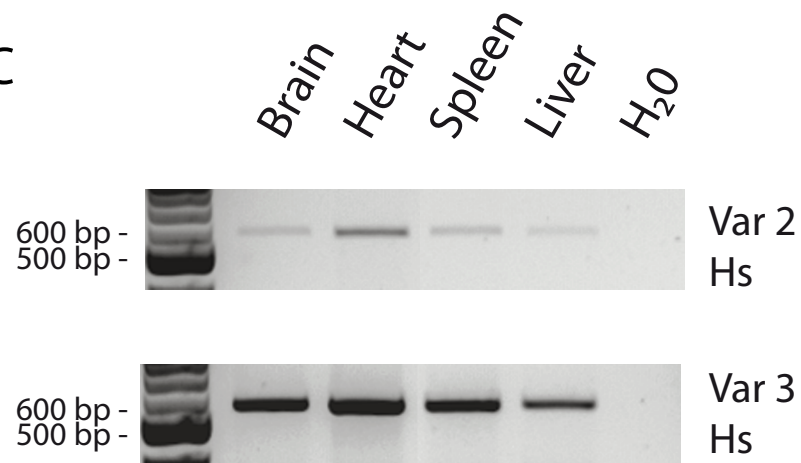

B

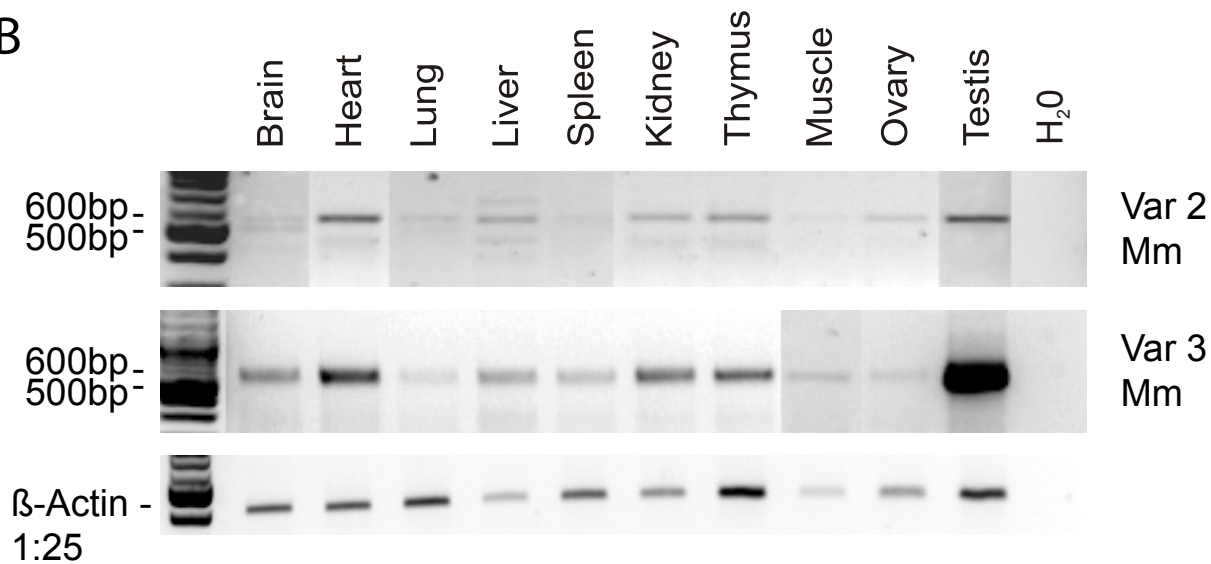

Supplement: Additional file 2 — Absence of nonsense-mediated decay in Jmjd6 splice variants. Two additional putative exons (green boxes) were identified (A). Alternative splicing using these two exons results in the generation of two additional transcripts of Jmjd6 (variants 2 and 3, respectively). Half arrows and orange lines in the schematic presentation of the transcripts highlight the combination of exons detected by RT-PCR in (B) and (C). Primers were designed to bind to the respective exons shown. (B) Experimental validation of the two new predicted alternative Jmjd6 transcripts using RT-PCR and agarose gel electrophoresis. Expression analyses confirmed that both splice variants are not subject to nonsense-mediated decay in adult mouse organs and (C) human tissues. Amplification of the housekeeping β-actin gene was used as a RNA loading control. [file 1471-2164-9-293-S2.pdf]

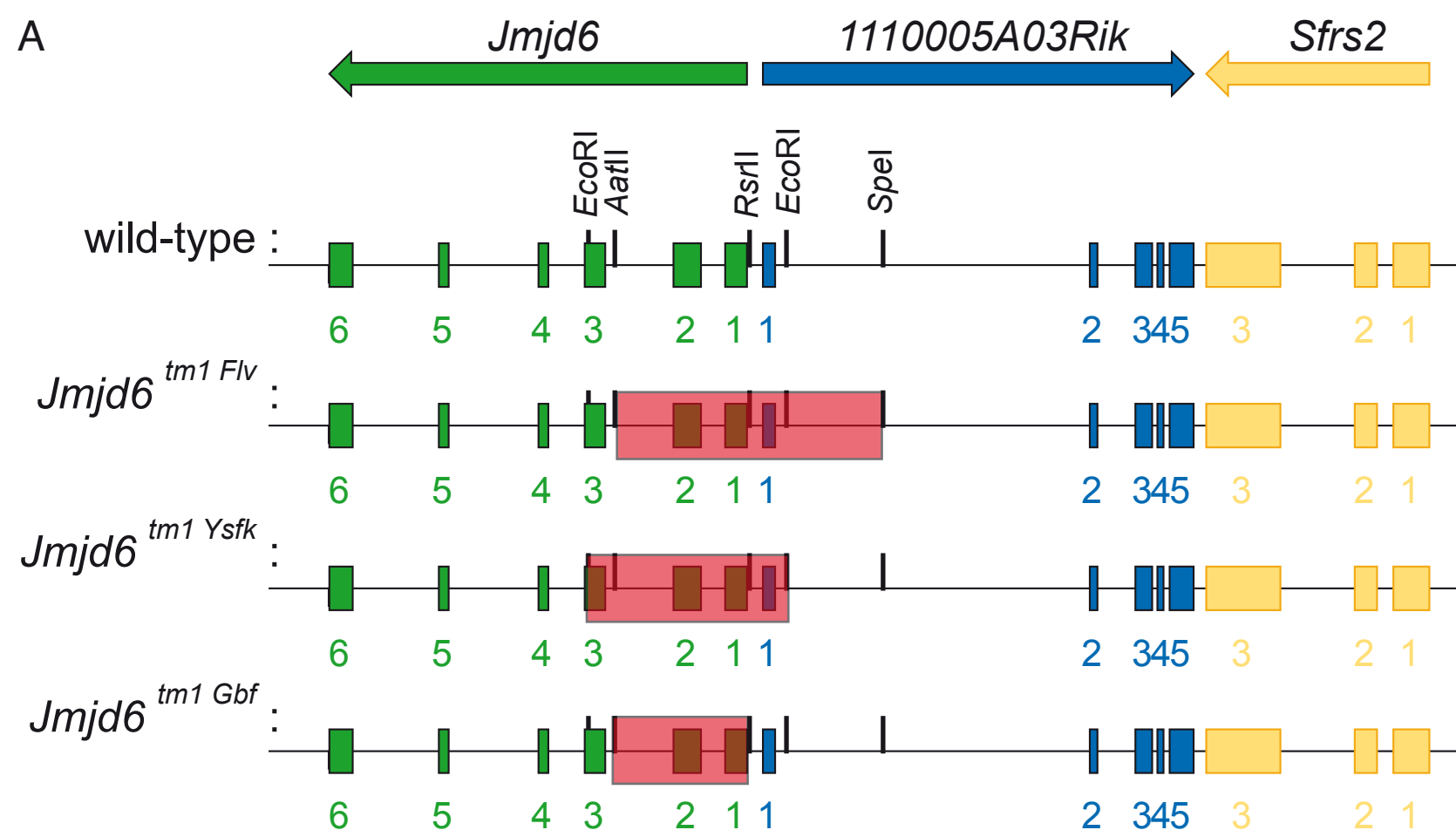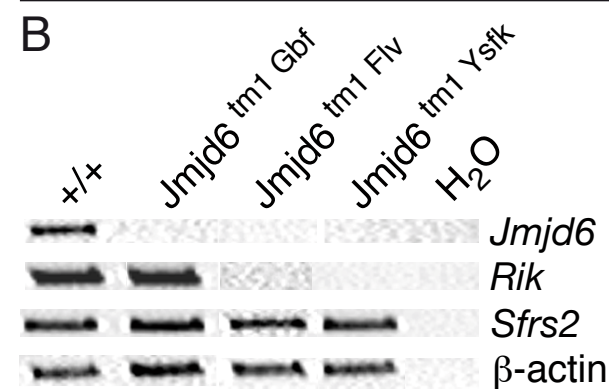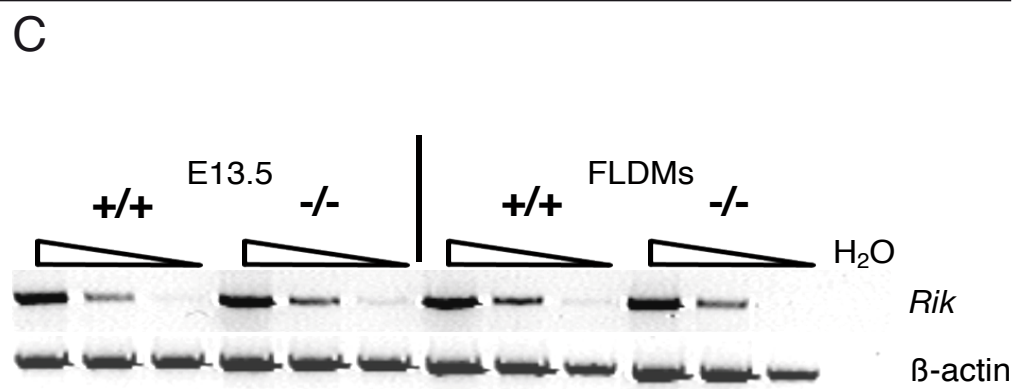

Supplement: Additional file 3 — Comparison of strategies used to inactivate the Jmjd6 gene in mouse ES-cells. Schematic representation of the mouse locus according to the Ensembl annotation of the Jmjd6 gene and its neighbouring genes (A). Arrows indicate transcriptional orientation of the genes along chromosome 11, from centromer to telomer. Jmjd6 exons are shown in green, exons of 1110005A03Rik in blue, and of Sfrs2 in yellow. Individual exons are numbered. The strategies used to generate the different targeted knock out alleles are shown. Red boxes indicate gene regions deleted by homologous recombination. The Jmjd6tm1Flv allele was generated by replacing an AatII/SpeI fragment by a neomycin resistance cassette. In the Jmjd6tm1Ysfk allele the EcoRI fragment and in the Jmjd6tm1Gbf allele the AatII/RsrII fragment were replaced respectively. Based on these chosen fragments, targeted inactivation of Jmjd6 seems to affect in two of the three alleles the neighbouring 1110005A03Rik locus. (B) RT-PCR analysis of the expression of the Jmjd6, 1110005A03Rik (Rik) and Sfrs2 genes in Jmjd6 wild type and Jmjd6 homozygous mutant embryos. The expression of 1110005A03Rik is not detectable in the Jmjd6tm1Flv allele and in the Jmjd6tm1Ysfk allele, whereas expression of Sfrs2 is unaltered in all three mouse lines investigated (C) Semi-quantitative RT-PCR analysis of 1110005A03Rik expression in Jmjd6tm1Gbf wild type and homozygous mutant embryos (embryonic stage E13.5) and in fetal-liver derived macrophages shows that the level of expression is not altered due to the targeted inactivation of the Jmjd6 gene in this mouse line. RT-PCR expression analysis was performed in differential dilution steps of cDNA material (1, 1:10, 1:100). In all RT-PCR expression experiments amplification of the housekeeping gene β-actin severed as loading control. [file 1471-2164-9-293-S3.pdf]

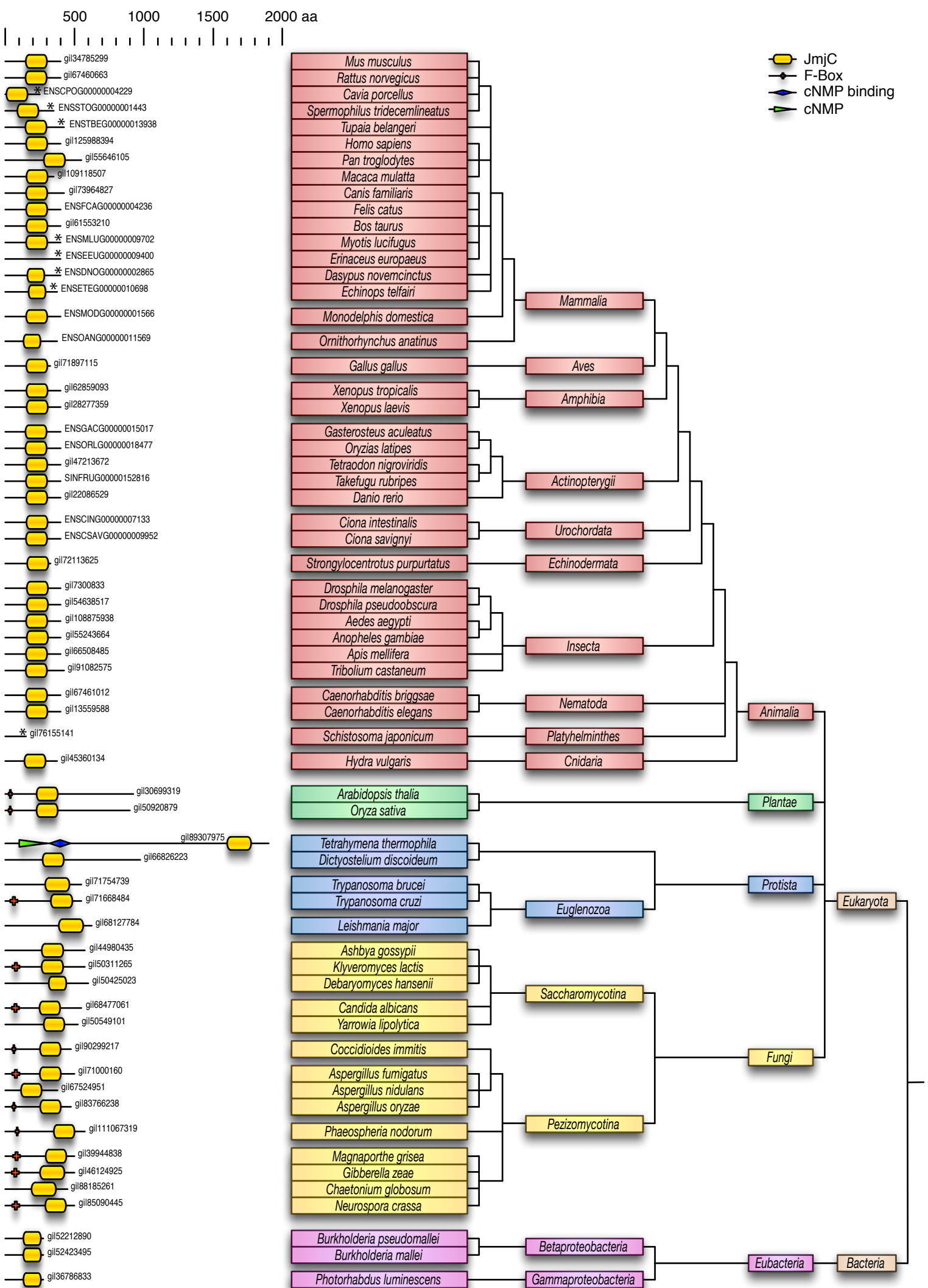

Supplement: Additional file 4 — Phylogenetic tree of live for identified putative Jmjd6 orthologs and their domain composition. Scientific classification of species with identified putative Jmjd6 orthologs was performed according to the NCBI taxonomy browser [108]. Schematic presentation of the grouping and categorisation of 62 species with identified Jmjd6 proteins. The individual species are shown on the left side, underlying colours highlight the kingdoms – animalia (red), plantae (green), protista (blue), fungi (yellow), and eubacteria (magenta), boxes on the right side represent the domain. The intermediate boxes show informative higher order ranks (e.g. genus, family, order, class, or phyla). On the left side, the domain composition for each protein is given. Length of polypeptides in amino acids (aa) are indicated on the top. Asterisks indicate partial or truncated protein sequences. [file 1471-2164-9-293-S4.pdf]

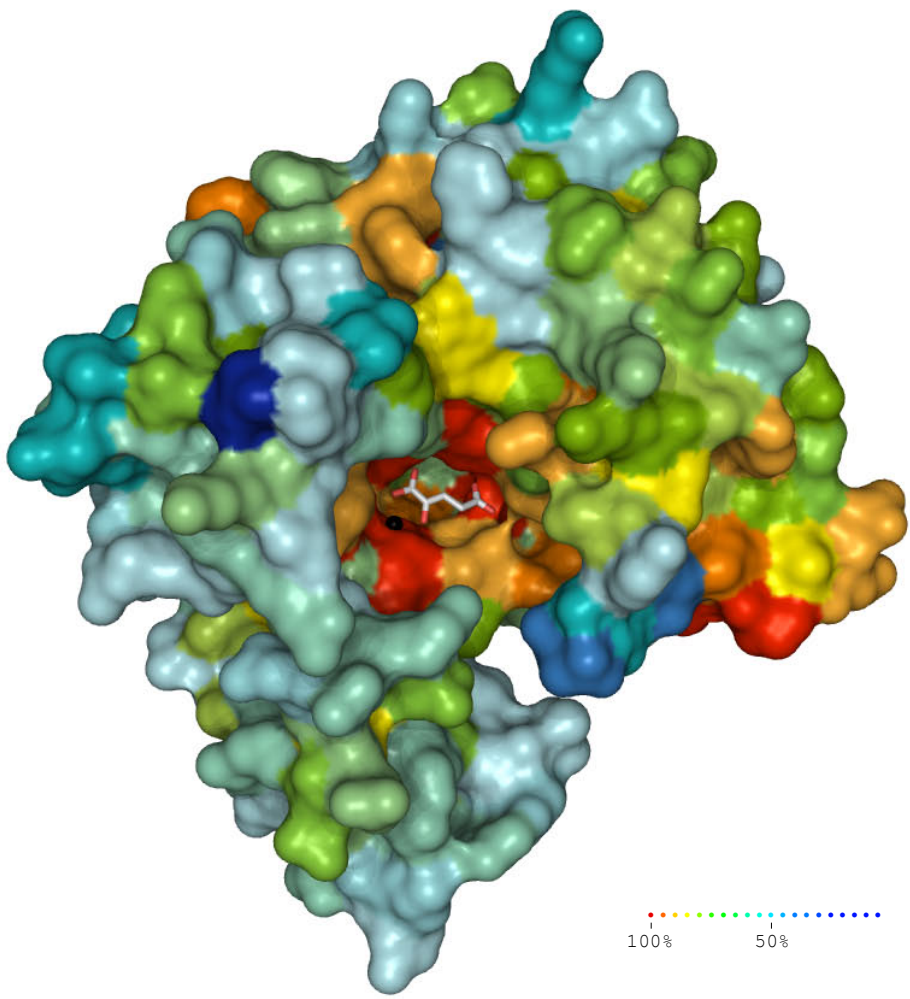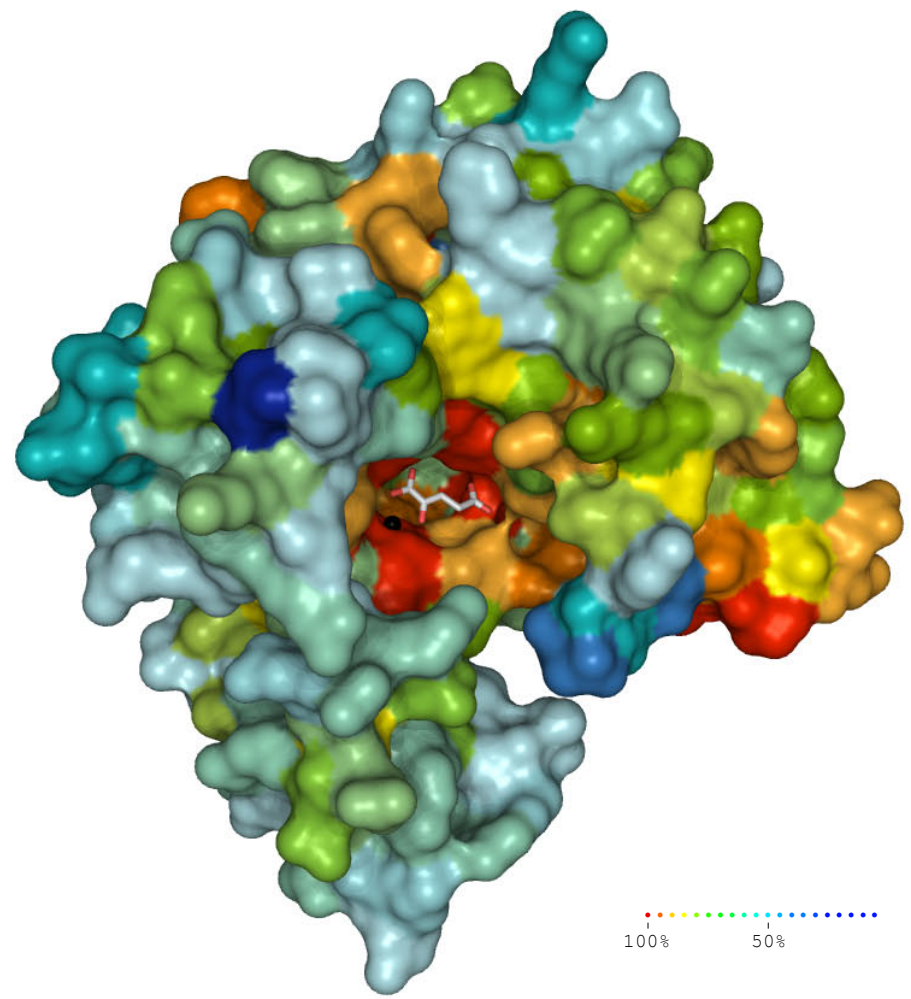

Supplement: Additional file 5 — Evolutionary conservation of surface exposed residues in the Jmjd6 protein model (stereoview). The surface model was computed using PyMol and coloured according to the sequence conservation code below in percent sequence identity in 54 analysed species. The coordinating Fe(II) of the catalytic triad is depicted in black, the co-substrate 2OG is shown as stick representation. [file 1471-2164-9-293-S5.pdf]
