# Supplementary material for: Genomic structure and expression of Jmjd6 and evolutionary analysis in the context of related JmjC domain containing proteins
Source: BMC Genomics. 2008 Jun 18;9:293. doi: 10.1186/1471-2164-9-293 (PMC2453528; doi:10.1186/1471-2164-9-293)
Supplement: Additional file 7 — Features of structurally resolved 2OG-Fe(II)-dependent dioxygenases in comparison to Jmjd6. Table describing features of structurally resolved and experimentally characterised 2OG-Fe(II)-dependent dioxygenases in comparison to Jmjd6. [file 1471-2164-9-293-S7.pdf]

**Features of structurally resolved 2OG-Fe(II)-dependent dioxygenases in comparison to Jmjd6**

| Protein | Major ß-sheets | Minor ß-sheets | Iron chelating aa * | 2-OG binding aa ** | pdb entry |
|---|---|---|---|---|---|
| alkB | ß 1 : Ala 116 - Tyr 122<br>ß 3 : Ile 143 - Gly 149<br>ß 6 : Val 175 - Trp 178<br>ß 8 : Arg 204 - Phe 209 | ß 2 : Leu 128 - His 131<br>ß 4 : Ala 152 - Phe 156<br>ß 5 : Lys 166 - Leu 170<br>ß 7 : His 187 - Ile 189 | His 131<br>Asp 133<br>His 187 | Tyr 122<br>Arg 204<br>Asn 206 | 2FD8 |
| ABH3 | ß 1 : Ser 175 - Tyr 181<br>ß 3 : Ile 204 - Gly 210<br>ß 6 : Leu 244 - Gly 249<br>ß 8 : Arg 269 - Phe 274 | ß 2 : Val 188 - His 191<br>ß 4 : Arg 213 - Lys 219<br>ß 5 : Arg 234 - Leu 239<br>ß 7 : Trp 255 - Val 259 | His 191<br>Asp 193<br>His 257 | Tyr 181<br>Arg 269<br>Asn 271 | 2IUW |
| PHD2 | ß 1 : Ala 298 - Tyr 303<br>ß 3 : Arg 322 - Tyr 329<br>ß 6 : Arg 362 - Trp 367<br>ß 8 : Arg 383 - Asp 392 | ß 2 : Tyr 310 - His 313<br>ß 4 : Leu 343 - Ile 345<br>ß 5 : Ala 354 - Ile 356<br>ß 7 : His 374 - Val 376 | His 313<br>Asp 315<br>His 374 | Tyr 303<br>Tyr 329<br>Arg 383 | 2G1M |
| PAHX | ß 1 : Ile 151 - Lys 161<br>ß 3 : Ile 189 - Ala 195<br>ß 6 : Thr 255 - Phe 256<br>ß 8 : Arg 275 - Ser 284 | ß 2 : (165-172)<br>ß 4 : Leu 206 - Val 208<br>ß 5 : Val 246 - Leu 248<br>ß 7 : His 264 - Ser 266 | His 175<br>Asp 177<br>His 264 | Lys 120<br>Ser 266<br>Arg 275 | 2A1X |
| P4H | n.d. | n.d. | His 412<br>Asp 414<br>His 483 | Lys 493<br>His 501 | |
| Hif1an | ß 1 : Leu 186 - Gly 190<br>ß 3 : Gln 203 - Lys 211<br>ß 6 : Val 270 - Ile 273<br>ß 8 : Thr 290 - Lys 298 | ß 2 : Val 195 - His 199<br>ß 4 : Lys 214 - Phe 219<br>ß 5 : Gly 260 - Val 265<br>ß 7 : Trp 278 - Ser 283 | His 199<br>Asp 201<br>His 279 | Tyr 145<br>Thr 196<br>Lys 214 | 1H2K |
| Jmjd2a | ß 1 : Tyr 175 - Gly 179<br>ß 3 : Tyr 195 - Gly 203<br>ß 6 : Phe 267 - Thr 270<br>ß 8 : Asn 284 - Phe 291 | ß 2 : Ser 184 - His 188<br>ß 4 : Lys 206 - Val 211<br>ß 5 : Asp 258 - Gln 262<br>ß 7 : Tyr 275 - Asn 280 | His 188<br>Glu 190<br>His 276 | Tyr 132<br>Asn 198<br>Lys 206 | 2GP3 |
| | | | | | |
| Jmjd6 | ß 1 : Arg 173 - Gly 178<br>ß 3 : Ser 194 - Val 200<br>ß 6 : Glu 263 - Val 267<br>ß 8 : Thr 281 - Phe 288 | ß 2 : Ser 182 - Ile 188<br>ß 4 : His 203 - Phe 209<br>ß 5 : Leu 255 - Gln 259<br>ß 7 : Trp 272 - Leu 278 | His 187<br>Asp 189<br>His 273 | Trp 174<br>Asn 197<br>Lys 204 | |

* red and orange coloured amino acids (aa) indicate $HxDx_nH$ or $HxEx_nH$ motifs, ** green and blue aa indicate Arg or Lys 2OG-binding residues
