# Supplementary material for: Genomic structure and expression of Jmjd6 and evolutionary analysis in the context of related JmjC domain containing proteins
Source: BMC Genomics. 2008 Jun 18;9:293. doi: 10.1186/1471-2164-9-293 (PMC2453528; doi:10.1186/1471-2164-9-293)
Supplement: Additional file 6 — Phylogenetic relationship of Jmjd6 to the superfamily of JmjC-domain containing proteins. In total 313 sequences of DSBH/JmjC domain containing proteins from humans and model organisms (mouse, zebrafish, pufferfish, nematode, fruit fly, and yeast) were analysed for their relationship to different subgroups of JmjC domain containing proteins. JmjC domain sequences of the proteins were analysed by multiple sequence alignment and subsequent bootstrap analysis using ClustalW (1000 bootstrap trials, 111 seeds) and PHYLIP (ProML). The resulting unrouted tree based on sequence similarity within the JmjC domain revealed 25 evolutionary conserved protein subgroups. These are highlighted in different colours according to the colour code on the left side. Five proteins that could not be grouped into subgroups are indicated in red letters. H.s. = Homo sapiens, M.m. = Mus musculus, D.r. = Danio rerio, T.n. = Tetraodon nigroviridis, T.r. = Takifugu rubripes, D.m. = Drosophila melanogaster, C.e. = Caenorhabditis elegans, S.c. = Saccharomyces cerevisiae, S.p. = Schizosaccharomyces pombe. [file 1471-2164-9-293-S6.pdf]

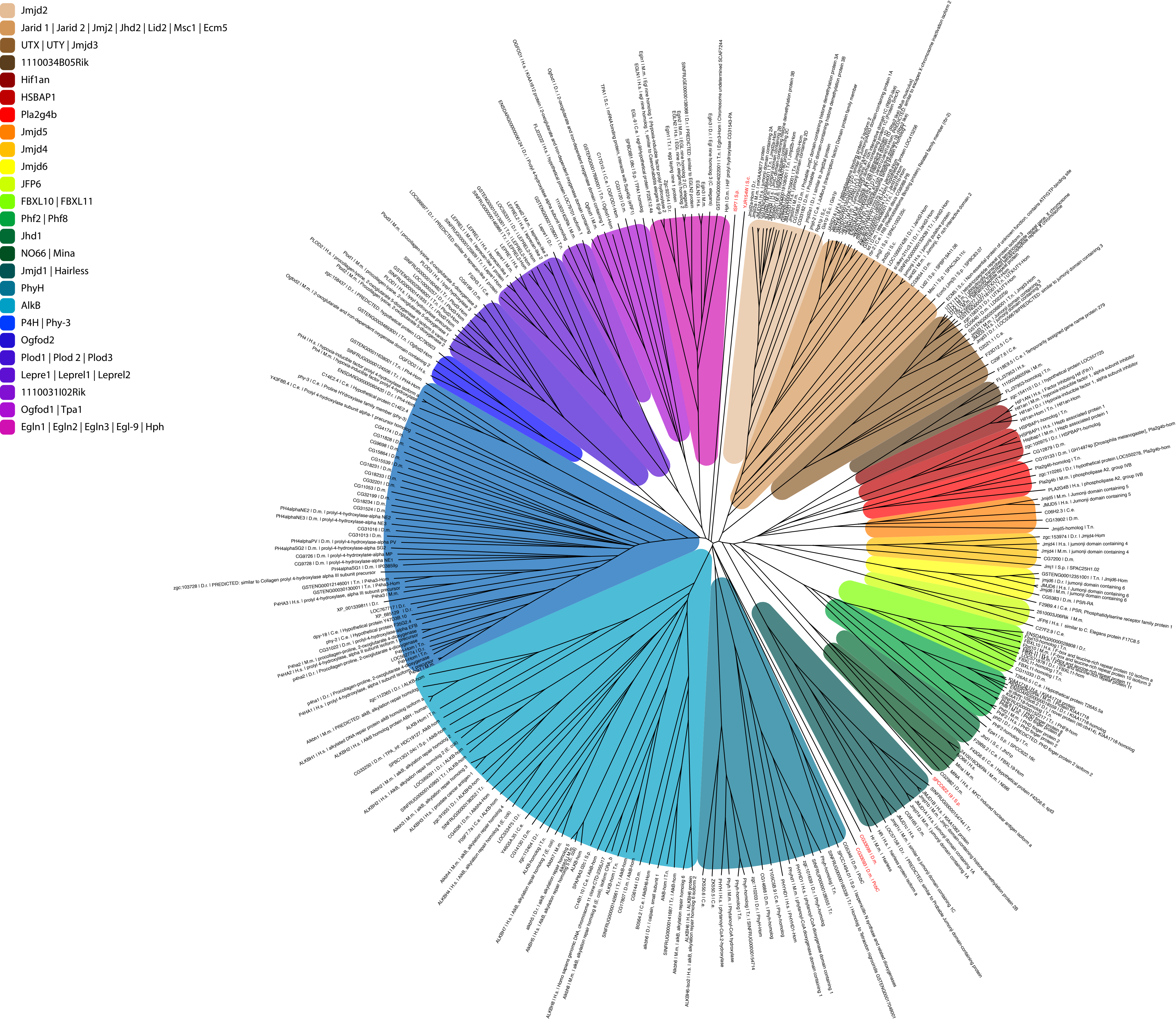
Jmjd2
Jarid 1 | Jarid 2 | Jmj2 | Jhd2 | Lid2 | Msc1 | Ecm5
UTX | UTY | Jmjd3
1110034B05Rik
Hif1an
HSBAP1
Pla2g4b
Jmjd5
Jmjd4
Jmjd6
JFP6
FBXL10 | FBXL11
Phf2 | Phf8
Jhd1
NO66 | Mina
Jmjd1 | Hairless
PhyH
AlkB
P4H | Phy-3
Ogfod2
Plod1 | Plod 2 | Plod3
Lepre1 | Leprel1 | Leprel2
1110031I02Rik
Ogfod1 | Tpa1
Egln1 | Egln2 | Egln3 | Egl-9 | Hph
