# Supplementary material for: Genomic structure and expression of Jmjd6 and evolutionary analysis in the context of related JmjC domain containing proteins
Source: BMC Genomics. 2008 Jun 18;9:293. doi: 10.1186/1471-2164-9-293 (PMC2453528; doi:10.1186/1471-2164-9-293)
Supplement: Additional file 1 — Validation of JMJD6 splices variants in humans. RT-PCR expression analysis of JMJD6 splice variants in a human cDNA tissue panel obtained from the BioChain Institute (Hayward, USA). Arrows indicate amplicons from correctly predicted splice variants that were confirmed by sequencing. Multiplex PCR amplification results in one verified unspecific PCR fragment migrating at approximately 400 bp. Amplification of the housekeeping gene Ribosomal Protein 9 (RPS9) was used as loading control. [file 1471-2164-9-293-S1.pdf]

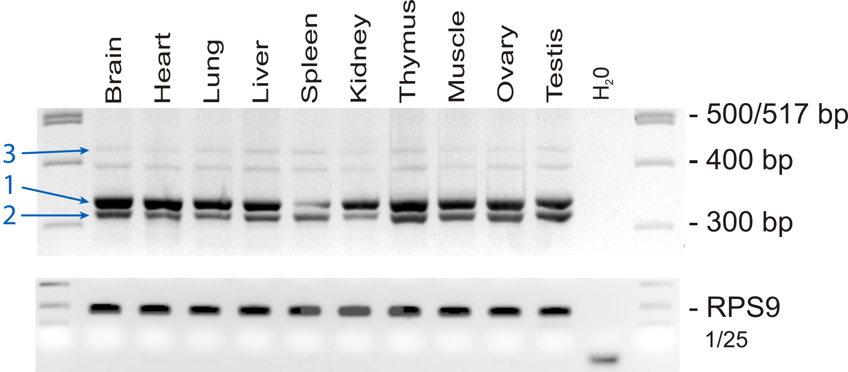
Brain
Heart
Lung
Liver
Spleen
Kidney
Thymus
Muscle
Ovary
Testis
H_20
- 500/517 bp
- 400 bp
- 300 bp
3
1
2
- RPS9
1/25
